# Autonomic function predicts cognitive decline in mild cognitive impairment: Evidence from power spectral analysis of heart rate variability in a longitudinal study

**DOI:** 10.3389/fnagi.2022.886023

**Published:** 2022-09-15

**Authors:** Paola Nicolini, Tiziano Lucchi, Carlo Abbate, Silvia Inglese, Emanuele Tomasini, Daniela Mari, Paolo D. Rossi, Marco Vicenzi

**Affiliations:** ^1^Geriatric Unit, Internal Medicine Department, Fondazione IRCCS Ca’ Granda Ospedale Maggiore Policlinico, Milan, Italy; ^2^IRCCS Fondazione Don Carlo Gnocchi, Milan, Italy; ^3^Department of Clinical Sciences and Community Health, University of Milan, Milan, Italy; ^4^Dyspnea Lab, Department of Clinical Sciences and Community Health, University of Milan, Milan, Italy; ^5^Cardiovascular Disease Unit, Internal Medicine Department, Fondazione IRCCS Ca’ Granda Ospedale Maggiore Policlinico, Milan, Italy

**Keywords:** autonomic nervous system, cognition, episodic memory, executive function, heart rate variability, mild cognitive impairment, longitudinal study, older adults

## Abstract

**Background:**

Despite the emerging clinical relevance of heart rate variability (HRV) as a potential biomarker of cognitive decline and as a candidate target for intervention, there is a dearth of research on the prospective relationship between HRV and cognitive change. In particular, no study has addressed this issue in subjects with a diagnosis of cognitive status including cognitive impairment.

**Objective:**

To investigate HRV as a predictor of cognitive decline in subjects with normal cognition (NC) or Mild Cognitive Impairment (MCI). Specifically, we tested the literature-based hypothesis that the HRV response to different physical challenges would predict decline in different cognitive domains.

**Methods:**

This longitudinal study represents the approximately 3-year follow-up of a previous cross-sectional study enrolling 80 older outpatients (aged ≥ 65). At baseline, power spectral analysis of HRV was performed on five-minute electrocardiographic recordings at rest and during a sympathetic (active standing) and a parasympathetic (paced breathing) challenge. We focused on normalized HRV measures [normalized low frequency power (LFn) and the low frequency to high frequency power ratio (LF/HF)] and on their dynamic response from rest to challenge (Δ HRV). Extensive neuropsychological testing was used to diagnose cognitive status at baseline and to evaluate cognitive change over the follow-up *via* annualized changes in cognitive Z-scores. The association between Δ HRV and cognitive change was explored by means of linear regression, unadjusted and adjusted for potential confounders.

**Results:**

In subjects diagnosed with MCI at baseline a greater response to a sympathetic challenge predicted a greater decline in episodic memory [adjusted model: Δ LFn, standardized regression coefficient (β) = −0.528, *p* = 0.019; Δ LF/HF, β = −0.643, *p* = 0.001] whereas a greater response to a parasympathetic challenge predicted a lesser decline in executive functioning (adjusted model: Δ LFn, β = −0.716, *p* < 0.001; Δ LF/HF, β = −0.935, *p* < 0.001).

**Conclusion:**

Our findings provide novel insight into the link between HRV and cognition in MCI. They contribute to a better understanding of the heart-brain connection, but will require replication in larger cohorts.

## Introduction

Mild cognitive impairment (MCI) is a transitional state lying along the continuum between normal cognitive aging and dementia (Petersen et al., 2018). It is characterized by slight cognitive deficits with no or minimal impairment in the activities of daily living (Petersen et al., 2018). MCI is emerging as a major health issue because it is considered to be the prodromal stage of dementia (Petersen et al., 2018) and because it will affect an ever-growing number of individuals. In fact, its prevalence increases with age (Petersen et al., 2018) and is bound to soar with the exponential aging of the population [United Nations [UN], and Department of Economic and Social Affairs Population Division [DESA], 2020]. In Italy, the prevalence of MCI has been reported to reach 34% in those over 90 (Pioggiosi et al., 2006).

Heart rate variability (HRV) is the physiological phenomenon by which the heart rate (HR) changes from beat to beat, producing fluctuations in the time intervals between adjacent R waves (RR intervals) on an electrocardiographic (ECG) recording (Malik et al., 1996; Laborde et al., 2017; Shaffer and Ginsberg, 2017). HRV reflects the influence on sinus node activity of the two branches of the autonomic nervous system (ANS)—sympathetic and parasympathetic (Malik et al., 1996; Laborde et al., 2017; Shaffer and Ginsberg, 2017)—and HRV analysis therefore provides a simple and reliable method for the assessment of autonomic function in clinical research (Nicolini et al., 2012).

An increasing number of cross-sectional studies have indicated an association between cognition and HRV. They have enrolled older subjects with neurocognitive disorders (for reviews see Cheng et al., 2020; Liu et al., 2022) or generally healthy individuals across the age spectrum [for a review see Forte et al. (2019a)], more seldom within the geriatric age range (e.g., Dalise et al., 2020).

The link between cognition and HRV appears to be related to their common neural substrate which is the Central Autonomic Network (CAN). The CAN is a complex network of central nervous system regions that are implicated in both cognitive processing and in the autonomic regulation of cardiovascular function (Thayer et al., 2009; Silvani et al., 2016). It encompasses brain structures such as the hippocampus, insula, locus coeruleus and prefrontal cortex, which project to preganglionic neurons of the sympathetic and parasympathetic nervous system. Thus, the CAN represents the neuroanatomical correlate of the brain-heart axis (Silvani et al., 2016). Also, autonomic dysfunction can lead to blood pressure (BP) dysregulation (Julius and Weder, 1989) which can contribute to cognitive impairment *via* cerebral hypoperfusion (Forte et al., 2019b; Jia et al., 2021).

The clinical relevance of the connection between cognition and HRV lies in the fact that HRV could serve as a potential biomarker of cognitive decline as well as a candidate target for intervention. There is a growing recognition that dementing illnesses follow a chronic disease model in which cognitive symptoms emerge late in the course of the disease process and are predated by pathological changes and/or neural dysfunction occurring several years before (Tan et al., 2014). Since traditional cerebrospinal fluid (CSF) and neuroimaging biomarkers are costly and/or invasive and not widely accessible or applicable (Tan et al., 2014), over the last decade particular momentum has been gained by measures of brain activity (Cassani et al., 2018; Engedal et al., 2020). Although data on HRV is still sparse and novel, it is acknowledged that HRV reflects a state of the brain (Ernst, 2017) and as such it is believed to hold considerable promise as an early biomarker of cognitive impairment (Forte et al., 2019a). Also, within the context of scant and controversial pharmacological treatment for MCI (Petersen et al., 2018; Chen et al., 2021; Tampi et al., 2021), HRV-biofeedback is a convenient and increasingly popular method of enhancing HRV (Lehrer and Gevirtz, 2014; Lehrer et al., 2020) with beneficial effects on cognitive performance across healthy and pathological populations of different ages (Lehrer et al., 2020; Tinello et al., 2021).

Nonetheless, to the best of our knowledge, no study has so far addressed the question of whether HRV can predict cognitive decline in subjects with cognitive impairment or has differentiated between individuals with normal cognition (NC) and MCI. As far as we are aware, there are only six studies in the literature that have investigated the longitudinal association between HRV and cognitive functioning (Britton et al., 2008; Mahinrad et al., 2016; Zeki Al Hazzouri et al., 2017; Knight et al., 2020; Schaich et al., 2020; Costa et al., 2021). Only one has exclusively involved older adults (Mahinrad et al., 2016) and none have included a diagnosis of cognitive status. They are all secondary analyses of large-scale epidemiological studies carried out for other purposes and therefore, despite their large sample sizes, they may suffer from some limitations. These comprise the use of routine ECG recordings and limited cognitive batteries, the lack of cognitive data at baseline, and the paucity of findings concerning episodic memory, and are described at length in the Supplementary Introduction.

In an attempt to fill this gap in the literature we report the findings from the 3-year cognitive follow-up of NC and MCI subjects aged ≥ 65 who were enrolled in a previous cross-sectional study on HRV and underwent HRV analysis in resting conditions and during a sympathetic and parasympathetic challenge. The study details have been described elsewhere (Nicolini et al., 2014) and will be recapitulated in the “Baseline assessment” section of the Methods.

We hypothesized that the autonomic response to the two challenges would predict decline in different cognitive domains. In particular, we supposed that the response to the sympathetic challenge would negatively predict decline in episodic memory whereas the response to the parasympathetic challenge would negatively predict decline in executive functioning.

Our assumptions were based on diverse lines of evidence from the literature that have shown that different components of the CAN are differentially involved in cognitive processing and in sympathetic versus parasympathetic autonomic control. Specifically, the hippocampus/parahippocampus, insula and locus coeruleus have been demonstrated to play a role in episodic memory and to generate sympathetic outflow, while the prefrontal cortex has been proved to be responsible for executive functioning and for parasympathetic activity. The evidence supporting these site-specific functions of the CAN is extensively reviewed in the Supplementary Introduction. Accordingly, within the more general relationship between cognition and HRV, several cross-sectional studies have found episodic memory to positively correlate with sympathetic HRV (Frewen et al., 2013; Nicolini et al., 2020; Hilgarter et al., 2021) and negatively correlate with parasympathetic HRV (Vasudev et al., 2012; Kim et al., 2018), and a number of investigators have noted a positive association between executive functions and parasympathetic HRV, both cross-sectionally (e.g., Forte et al., 2019a) and longitudinally (Mahinrad et al., 2016; Zeki Al Hazzouri et al., 2017; Knight et al., 2020; Schaich et al., 2020; Costa et al., 2021).

## Materials and methods

### Baseline assessment

#### Study population

We considered for inclusion 475 community-dwelling older subjects (aged ≥ 65) who consecutively attended a first geriatric visit at the Geriatric Outpatient Unit of our university hospital from January to December 2012. Referrals were made by general practitioners for a wide spectrum of age-related health problems. Eighty subjects with a known diagnosis of dementia were excluded. Of the remaining 395 subjects, 117 were eligible based on a number of exclusion criteria and were invited to undergo neuropsychological testing (see following sections). Of the 113 subjects who consented to neuropsychological testing, 23 were diagnosed with dementia and excluded, leaving 90 subjects with a diagnosis of NC or MCI. Of these, 5 declined further participation, so that 85 subjects (*n* = 44 NC and *n* = 41 MCI) were asked to take part in the subsequent clinical and autonomic assessment, which was carried out within 1 month from the neuropsychological assessment. Of the 85 participants, 2 were excluded during the autonomic assessment (*n* = 1 baseline respiratory rate < 9 breaths/min, *n* = 1 postural vasovagal reaction) and 3 during the HRV analysis (*n* = 1 paroxysmal supraventricular tachycardia, *n* = 2 excessive ectopic beats). Thus, the study ultimately enrolled 80 subjects, 40 with NC and 40 with MCI.

#### Exclusion criteria

Exclusion criteria were conditions precluding HRV analysis as well as diseases and medications with an established and significant effect on HRV. They have been extensively accounted for and referenced in our previous work (Nicolini et al., 2014) and were as follows: (a) non-sinus rhythm (atrial fibrillation and other arrhythmias, paced rhythms), (b) heart disease (coronary artery disease, heart failure), diabetes mellitus, neurological and psychiatric diseases (Parkinson’s disease, stroke, major depression) and severe diseases (respiratory, renal, autoimmune and neoplastic), (c) cardioactive medications: beta-blockers, alpha-blockers, centrally-acting calcium-channel blockers, class I and III antiarrhytmic drugs, digoxin, (d) psychotropic medications: tricyclic antidepressants, selective serotonin-noradrenaline reuptake inhibitors, atypical antidepressants, antipsychotics and cholinesterase inhibitors.

#### Neuropsychological assessment

The neuropsychological assessment was performed by means of a comprehensive battery of tests covering different cognitive domains: episodic memory, executive functioning, language, visuospatial skills and ideomotor praxis. The neuropsychological tests and their references can be found in Supplementary Table 1. MCI was diagnosed according to consensus criteria of objective cognitive impairment on neuropsychological testing, essentially preserved daily functioning [i.e., intact basic activities of daily living (BADL) with no or minimal impairment of instrumental activities of daily living (IADL)] and no dementia (Petersen et al., 2018).

Raw neuropsychological test scores were converted to age-, sex- and education-adjusted scores based on published normative data for the Italian population and objective cognitive impairment was defined as having an adjusted score in at least one neuropsychological test below the 10th percentile of the normative score distribution. We selected the 10th percentile threshold, in accordance with several other Authors (e.g., Solfrizzi et al., 2004; Delano-Wood et al., 2009), because we believe that, relative to the 1 and 1.5 standard deviation cut-offs (i.e., the 7th and 16th percentiles), also commonly accepted (Albert et al., 2011), it provides a more appropriate balance between the risk of under- and over-diagnosing MCI.

#### Clinical assessment

During the clinical assessment we collected information on sociodemographics (age, sex, education), anthropometrics (Body Mass Index), lifestyle habits (alcohol and coffee consumption, physical activity), blood tests for vascular risk (glucose and lipid panel), history of hypertension (defined as current antihypertensive drug therapy), medication history, psychological symptoms [short Geriatric Depression Scale (GDS-s) and State-Trait Personality Inventory-trait anxiety subscale (STPI-TA)] and comorbidity [Cumulative Illness Rating Scale-comorbidity (CIRS-m)]. Functional status was assessed by means of the BADL and IADL scales. The Mini Mental State Examination (MMSE), corrected for age and education, was used to provide a crude measure of global cognitive functioning.

All measures used in the clinical assessment have been referenced in our previous work (Nicolini et al., 2014).

#### Autonomic assessment

The autonomic assessment was conducted in a quiet room, with dimmed lighting and a comfortable temperature (22–24°C) between 8:30 and 10:30 a.m. in order to minimize the effect of circadian changes in HRV. Participants were instructed to consume a light breakfast and refrain from caffeinated beverages, alcohol, smoking and vigorous physical activity in the 12 h prior to testing. Three-channel ECG recordings for HRV analysis were obtained by means of a digital Holter recorder (Spider View, Sorin Group Company).

The experimental protocol was composed of three stages: (1) supine rest with free breathing (resting condition): 15 min during which the subjects were asked to remain awake, silent and still, breathing spontaneously, (2) active standing (sympathetic challenge): 10 min in which the subjects were asked to remain still and silent, breathing spontaneously, after standing upright in as smooth a motion as possible, and (3) supine paced breathing at 12 breaths/minute (0.2 Hz) (parasympathetic challenge): 15 min during which the subjects breathed, as regularly as possible and at a “comfortable” tidal volume, in time to an electronic metronome set at 24 acoustic signals per minute (2.5 s inspiration, 2.5 s expiration). Given the nature of our study population, this stage was made as simple as possible: the first 10 min were devoted to familiarization with the breathing protocol and, throughout, the subjects were not asked to directly synchronize their breathing rhythm with the metronome but to follow voice indications from the experimenters. To allow for stabilization, only the last 5 min of each stage were analyzed. The spontaneous respiratory rate was visually monitored and subjects with a respiratory rate < 9 breaths/min (i.e., < 0.15 Hz) or > 24 breaths/min (i.e., > 0.40 Hz) were excluded from HRV analysis because, in these conditions, there is a shift of the high frequency power (HF) band that precludes proper interpretation of power spectral analysis (PSA) (Laborde et al., 2017).

#### Heart rate variability analysis

As far as HRV analysis was concerned, three methodological issues are worth noting. First, it was performed by PSA because the HRV Task Force guidelines (Malik et al., 1996) recommend that 5-min recordings be processed by frequency-domain methods, which are considered more sensitive (Malik and Camm, 1990), and 24-h recordings by time-domain methods, which have greater accuracy and predictive value over longer periods (Malik et al., 1996; Shaffer and Ginsberg, 2017). We employed commercial software (Synescope version 3.10, Sorin Group Company) which conducts PSA by means of the Fast Fourier Transform (FFT), after linear interpolation/resampling at 4 Hz of the discrete event series (to obtain a regularly time-sampled signal) and filtering with a Hanning window (to attenuate leakage effects). Although the software automatically detects non-sinus beats, the recordings were always manually overread by an experienced investigator, blinded to the subjects’ cognitive status, in order to ensure correct QRS complex classification and rhythm identification. Since there is no clear indication in the literature as to the amount of ectopic beats that it is acceptable to remove or remove and interpolate, we selected the most restrictive criterion of 1% of the total number of beats (Nicolini et al., 2014). Thus, recordings with excessive atrial or ventricular ectopic beats were excluded from analysis as were those with other arrhythmias.

Second, PSA can yield several different indices: total power (TP, ≈ ≤ 0.40 Hz), very low frequency power (VLF, ≤ 0.04 Hz), low frequency power (LF, 0.04–0.15 Hz), HF (0.15–0.40 Hz), normalized low frequency power [LFn = LF/(TP-VLF) × 100], normalized high frequency power [HFn = HF/(TP-VLF) × 100] and the low frequency power to high frequency power ratio (LF/HF). We chose to focus on LFn and LF/HF as markers of autonomic function because they can be considered indices of sympathetic activation/parasympathetic withdrawal (e.g., Malik et al., 1996, 2019; Montano et al., 2009) while other measures are less physiologically meaningful. TP only quantifies overall autonomic modulation (Malik et al., 1996). VLF is best assessed over 24 h (Malik et al., 1996; Shaffer and Ginsberg, 2017) and its interpretation is still uncertain (Shaffer and Ginsberg, 2017). The nature of LF is highly controversial and it has been viewed as reflecting prevalently sympathetic modulation (Malik et al., 1996), mixed sympathetic and parasympathetic modulation (Malik et al., 1996; Goldstein et al., 2011), and predominantly parasympathetic modulation (Reyes del Paso et al., 2013). Even if HF is an index of pararasympathetic modulation (Malik et al., 1996; Laborde et al., 2017; Shaffer and Ginsberg, 2017), there is evidence that HFn provides more accurate information on the state of the parasympathetic nervous system (Montano et al., 2009), also because HF is a more highly dispersed variable (Nunan et al., 2010). However, HFn was not taken into account because it is specularly correlated with LFn, which thus shares the same advantages (Montano et al., 2009; Nunan et al., 2010).

Third, we decided to concentrate on the changes of LFn and LF/HF from rest to challenge, i.e., Δ HRV = HRV challenge—HRV rest. This approach is based on the notion that Δ HRV indices are especially sensitive measures of autonomic modulation since they directly quantify the response to a stressor and explore the dynamic range of the ANS (Montano et al., 2009; Malik et al., 2019). Accordingly, much HRV literature uses Δ HRV indices (e.g., Niu et al., 2018; Suga et al., 2019; Knight et al., 2020) and studies in subjects with cognitive impairment by us (Nicolini et al., 2014, 2020) and others (e.g., Mellingsæter et al., 2015) have found differences in challenge-associated but not resting HRV. In particular, active standing Δ LFn and Δ LF/HF reflect sympathetic activation/parasympathetic withdrawal, while paced breathing Δ LFn and Δ LF/HF reflect parasympathetic activation/sympathetic withdrawal.

### Follow-up

After approximately 3 years from the baseline assessment, in order to evaluate cognitive change, participants were invited to attend a second neuropsychological evaluation using the same battery of neuropsychological tests employed at baseline. A geriatric assessment was also carried out with the same instruments used to measure functional and global cognitive status at baseline, i.e., the BADL, IADL and MMSE scales.

MCI was diagnosed as previously described. Dementia was diagnosed by means of standard criteria: the National Institute on Aging-Alzheimer’s association (NIA-AA) criteria for all-cause and Alzheimer’s disease (AD) dementia (McKhann et al., 2011) and the American Heart Association/American Stroke Association (AHA/ASA) criteria for vascular dementia (VAD) (Gorelick et al., 2011). Further details on the diagnosis of dementia are given in the Supplementary Materials and Methods.

It should be noted that, although a diagnosis of cognitive status was also made at follow-up, in our study the reference for the diagnosis of cognitive status was taken to be the same time of the HRV assessment, i.e., baseline. Since at baseline subjects with NC and MCI were enrolled in the study while subjects with dementia were excluded from it, our hypotheses were tested only in the two groups of subjects diagnosed with NC and MCI at baseline (also see Limitations).

The study was conducted in accordance with the Declaration of Helsinki and was approved by the ethics committee of the Fondazione IRCCS Ca’ Granda Ospedale Maggiore Policlinico in Milan, Italy. All participants gave written informed consent to participation in the study.

### Statistical analysis

Data are reported as mean (standard deviation) for continuous variables and as number (percentage) for categorical variables.

In the two cognitive groups at baseline (NC and MCI) the neuropsychological test scores were compared at baseline and follow-up by means of the paired *t*-test or the Wilcoxon sign-ranked test as appropriate.

Linear regression was performed in the NC and MCI groups at baseline to investigate if the autonomic response at baseline predicted cognitive change from baseline to follow-up. The annualized cognitive change score (see later) was taken as dependent variable while the independent variables were the Δ HRV indices (the variables of interest) as well as potential confounders which were selected *a priori* from the literature according to their being known risk factors for cognitive decline: socio-demographic factors (age, sex and education), physical activity and physical/mental comorbidity (e.g., Roberts and Knopman, 2013). Adjustment was also carried out for baseline cognition and resting HRV. Both simple (unadjusted) and multiple (adjusted) linear regression were conducted.

In accordance with the study hypotheses, two different regression models were fitted for each of the NC and MCI groups: one with the episodic memory change score as the outcome and the sympathetic Δ HRV indices (i.e., active standing Δ LFn and Δ LF/HF) among the predictors (model 1), the other with the executive functioning change score as the outcome and the parasympathetic Δ HRV indices (i.e., paced breathing Δ LFn and Δ LF/HF) among the predictors (model 2).

Composite scores were computed for episodic memory and executive functioning by means of a Z-score transformation. The raw scores of the individual neuropsychological tests were converted to Z-scores based on the mean and standard deviation of published normative data for the Italian population (see Supplementary Table 1) and then averaged to yield a domain-specific score. Scores quantifying response time (i.e., Trail-Making Tests A and B) or number or errors (i.e., Cognitive Estimates total and bizarre) were multiplied by −1 so that lower Z-scores consistently indicated poorer performance. We chose to use such composite cognitive scores because they offer several advantages: they minimize the likelihood of type I error associated with multiple testing, have greater reliability and reduce floor and ceiling effects (Morris et al., 1999; Williams et al., 2019). The cognitive change score for each domain was calculated by subtracting the baseline Z-score from the follow-up Z-score and it was then annualized by dividing it by the number of years of follow-up, so as to account for different follow-up durations among the study participants.

As far as comorbidity was concerned, with the aim of achieving a more parsimonious model, physical and mental comorbidities were combined to create an additive index. The physical burden of illness was measured by the CIRS-m score. Psychological distress was quantified by the GDS-s and STPI-TA scores. For comparability, all three scores were rescaled between 0 and 1 using a simple linear stretch according to the formula X’ (rescaled score) = [X (original score) – X min (minimum score value)]/[X max (maximum score value) – X min], and were then summed. The choice to standardize these measures to a common metric by a minimum-maximum normalization rather than a Z-score transformation, as was the case for the cognitive scores, is explained hereafter. First, normative neuropsychological test data for the Italian population are well-established and are the cornerstone of neuropsychological assessment [see e.g., Spinnler and Tognoni (1987) in Supplementary Table 1]. On the contrary, normative data for the CIRS-m, GDS-s and STPI-TA scores are either lacking (e.g., for the CIRS-m) or limited by representativeness issues [e.g., norms for the GDS-s are available for mentally and physically healthy older adults in New Zealand (Knight et al., 1983); norms for the STPI-TA are available for a different 10-item form of the STPI-TA in a French study (Bergua et al., 2016)]. Second, although the current study sample could have been taken as a reference for norming, we believed this would have been a less appropriate approach given its relatively small size. Nonetheless, if the three scales are aggregated by using a Z-score transformation based on our sample data, the pattern of significance is unaffected (see Supplementary Tables 2, 3).

In order to meet the assumptions of normality and/or homoscedasticity, two outliers (standardized residual > |3|) (Stevens, 2012) were omitted from the regression analyses: one in the NC group for model 2 and one in the MCI group for model 1. None of the omitted (or included) data points were influential (Cooke’s distance < 1) (Stevens, 2012). Indeed, retaining all data and repeating the analyses with non-parametric testing (i.e., Spearman’s simple and partial correlations) does not substantively change the results despite some expected loss of statistical power (Sedgwick, 2015; see Supplementary Tables 4, 5).

The regression models satisfied the assumptions of normality (Shapiro–Wilk’s test, *p* > 0.05), homoscedasticity (Breusch Pagan test, *p* > 0.05), independence of errors (Durbin Watson values between 1.5 and 2.5) and no significant multicollinearity [variance inflation factor (VIF) < 5] (Stevens, 2012).

Inflation of type I error due to multiple testing was controlled for by means of the Benjamini–Hochberg procedure (Benjamini and Hochberg, 1995) with the false discovery rate (FDR) set at the conventional level for alpha (5%).

Analyses were performed by means of the statistical packages SPSS version 25.0 (SPSS Inc., Chicago, IL, United States) and R version 3.5.2 (The R Foundation for Statistical Computing, Vienna, Austria) for Windows. A *P* value ≤ 0.05 was considered statistically significant.

Due to the novelty of the original study no power calculation was performed *a priori* (Nicolini et al., 2014). However, we conducted a power determination analysis for the current sample size with G*power (Faul et al., 2009) based on the data of Mahinrad et al. (2016) from a similarly aged population. Cohen’s d for differences in cognitive test scores (Stroop, Letter-Digits Coding and Picture-Word Learning total) between the high and low HRV tertiles (*d* = 2.7–3.1) was converted to Cohen’s f^2^ (f^2^ = 1.8–2.4) according to Cohen (1988) so as to obtain an effect size estimate for linear regression. Both measures indicated large effect sizes and, in order to be conservative, the lowest f^2^ value (f^2^ = 1.8) was used in the power analysis. The achieved sample sizes (*n* = 36 NC, *n* = 33 MCI) yielded an almost 100% power for multiple linear regression with a 5% alpha level (two tailed) and eight predictors.

## Results

The mean duration of the follow-up was 2.8 years (range 2.0–3.6 years). Of the 80 subjects enrolled at baseline (*n* = 40 NC and *n* = 40 MCI), 9 were lost to follow-up: 7 due to mortality (*n* = 2 NC and *n* = 5 MCI) and 2 due to refusal to participate further (*n* = 1 NC and *n* = 1 MCI). Thus, the analyses were performed on 71 subjects: 37 who were diagnosed with NC at baseline and 34 who were diagnosed with MCI at baseline. Over the follow-up, the 37 subjects with NC at baseline either remained NC (*n* = 25) or evolved to MCI (*n* = 11) or to dementia (*n* = 1), while the 34 subjects with MCI at baseline either remained MCI (*n* = 22) or reverted to NC (*n* = 1) or evolved to dementia (*n* = 11). The study sample at follow-up therefore included 26 subjects with NC, 33 subjects with MCI and 12 subjects with dementia. Among the 12 incident cases of dementia 9 were AD dementia and 3 VAD.

Tables 1, 2 show the neuropsychological test scores at baseline and follow-up in subjects with a baseline diagnosis of NC and MCI respectively. As expected, there was a decline in cognitive performance over time.

**TABLE 1:** Neuropsychological test scores at baseline and follow-up in subjects with NC at baseline (*n* = 37).

Test	Baseline	Follow-up	*P*-value
**Episodic memory**			
Prose recall	13.3 (2.3)	12.0 (3.1)	**0.021** ^a^
ROCF-delayed recall	23.4 (5.3)	20.5 (7.0)	0.066^a^
**Executive functions**			
Bell Test	34.4 (0.8)	33.9 (1.1)	**0.042** ^a^
Digit Cancellation Test	53.5 (5.0)	52.8 (6.4)	0.599^a^
Digit Span Forwards	5.7 (0.9)	5.6 (1.0)	0.827^b^
Digit Span Backwards	4.4 (0.6)	4.5 (0.8)	0.299^a^
Trail-Making Test A	28.8 (12.6)	30.8 (13.8)	0.561^b^
Trail-Making test B	58.5 (32.6)	74.8 (63.6)	0.451^b^
Weigl’s Test	12.2 (1.7)	11.4 (2.7)	0.122^b^
Cognitive Estimates-total	10.3 (1.5)	14.2 (3.3)	**< 0.001** ^a^
Cognitive Estimates-bizarre	1.6 (0.8)	3.5 (2.0)	**< 0.001** ^a^
Raven’s CPM	32.9 (3.8)	31.8 (5.4)	0.421^b^
Letter fluency	37.5 (7.6)	33.9 (8.9)	0.074^a^
**Language**			
Category fluency	18.6 (3.4)	18.5 (4.5)	0.777^b^
Picture naming	75.0 (2.8)	74.6 (4.3)	0.937^b^
Token Test	33.6 (1.1)	33.0 (2.8)	0.190^a^
**Visuospatial skills**			
ROCF-copy	34.9 (3.7)	35.0 (2.7)	0.899^b^
Copy of geometric figures	13.7 (0.4)	13.6 (0.8)	0.793^b^
**Ideomotor praxis**			
De Renzi’s test-right upper limb	71.6 (0.8)	71.3 (1.3)	0.210^b^
De Renzi’s test-left upper limb	71.0 (1.6)	71.4 (1.0)	0.142^b^

Neuropsychological test scores reported as mean (standard deviation) and demographically-adjusted. Higher scores indicate better cognitive performance except for the Trail-Making and Cognitive Estimates tests for which the reverse applies.

^a^Paired t-test, ^b^Wilcoxon signed-rank test. Significant results are shown in bold typeface. NC, Normal Cognition; ROCF, Rey-Osterrieth Complex Figure; CPM, Colored Progressive Matrices.

**TABLE 2:** Neuropsychological test scores at baseline and follow-up in subjects with MCI at baseline (*n* = 34).

Test	Baseline	Follow-up	*P*-value
**Episodic memory**			
Prose recall	8.1 (4.7)	7.8 (4.8)	0.656^a^
ROCF-delayed recall	14.3 (7.0)	11.9 (9.7)	**0.027** ^b^
**Executive functions**			
Bell Test	31.5 (2.7)	32.1 (3.5)	0.256^a^
Digit Cancellation Test	50.1 (5.7)	46.9 (8.7)	**0.017** ^b^
Digit Span Forwards	5.3 (0.9)	4.9 (1.4)	0.124^b^
Digit Span Backwards	3.7 (0.7)	3.6 (1.4)	0.774^b^
Trail-Making Test A	45.9 (23.8)	53.2 (45.7)	0.970^b^
Trail-Making test B	200.6 (136.8)	244.4 (169.1)	**0.008** ^b^
Weigl’s Test	9.4 (2.5)	8.6 (2.8)	0.088^a^
Cognitive Estimates-total	13.2 (2.3)	17.8 (3.4)	**< 0.001** ^a^
Cognitive Estimates-bizarre	2.6 (1.2)	5.1 (2.2)	**< 0.001** ^a^
Raven’s CPM	27.5 (4.7)	29.3 (16.1)	0.428^b^
Letter fluency	30.8 (8.5)	27.8 (10.3)	**0.034** ^a^
**Language**			
Category fluency	13.7 (3.1)	13.5 (4.1)	0.702^a^
Picture naming	70.1 (5.2)	67.1 (7.6)	**0.005** ^b^
Token Test	30.8 (2.0)	30.3 (2.8)	0.316^a^
**Visuospatial skills**			
ROCF-copy	32.1 (4.2)	29.1 (5.9)	**0.006** ^b^
Copy of geometric figures	12.4 (1.4)	12.0 (2.5)	0.889^b^
**Ideomotor praxis**			
De Renzi’s test-right upper limb	70.5 (1.8)	69.6 (3.6)	0.136^b^
De Renzi’s test-left upper limb	69.9 (1.9)	69.5 (3.6)	0.927^b^

Neuropsychological test scores reported as mean (standard deviation) and demographically-adjusted. Higher scores indicate better cognitive performance except for the Trail-Making and Cognitive Estimates tests for which the reverse applies.

^a^Paired t-test, ^b^Wilcoxon signed-rank test. Significant results are shown in bold typeface. MCI, Mild Cognitive Impairment; ROCF, Rey-Osterrieth Complex Figure; CPM, Colored Progressive Matrices.

Tables 3, 4 show the results of the linear regression in subjects with a baseline diagnosis of NC and MCI respectively. In the NC group Δ HRV indices were not found to be significant predictors of cognitive change; although the association between standing Δ LF/HF and episodic memory decline was significant in the unadjusted model [standardized regression coefficient (β) = −0.395, *p* = 0.016], it became non-significant after controlling for potential confounders (β = −0.148, *p* = 0.383). Instead, in the MCI group all Δ HRV indices were significant predictors of cognitive change in both unadjusted and adjusted models. In particular, after adjustment for potential confounders, a greater response to a sympathetic challenge predicted a greater decline in episodic memory (Δ LFn, β = −0.528, *p* = 0.019; Δ LF/HF, β = −0.643, *p* = 0.001) whereas a greater response to a parasympathetic challenge predicted a lesser decline in executive functioning (Δ LFn, β = −0.716, *p* < 0.001; Δ LF/HF, β = −0.935, *p* < 0.001).

**TABLE 3:** HRV indices as predictors of cognitive change in subjects with NC at baseline (*n* = 37).

	Unadjusted model^a^			Adjusted model^b^		
	β	*P*-value	*Q*-value^c^	β	*P*-value	*Q*-value^c^
**Active standing**						
Δ LFn (n.u)^†^	0.166	0.327	0.476	0.202	0.234	0.374
Δ LF/HF^†^	–0.395	**0.016**	**0.031**	–0.148	0.383	0.500
**Paced breathing**						
Δ LFn (n.u)^‡¶^	–0.086	0.617	0.672	0.098	0.630	0.672
Δ LF/HF^‡¶^	–0.143	0.406	0.500	0.071	0.786	0.786

^a^Simple linear regression with the Δ HRV index as independent variable and the annual change in the cognitive Z-score as dependent variable, ^b^Multiple linear regression adjusted for age, sex, education, physical activity, morbidity (additive index), resting HRV and baseline cognitive Z-score, ^c^P-value corrected for multiple testing by means of the Benjamini–Hochberg procedure with a 5% False Discovery Rate (FDR), ^†^Dependent variable: annual change in episodic memory Z-score (model 1), ^‡^Dependent variable: annual change in executive functioning Z-score (model 2), ^¶^One outlier removed from the analyses. Significant results are shown in bold typeface. NC, Normal Cognition; β, standardized regression coefficient; LFn, normalized low frequency power; n.u, normalized units; LF/HF, low frequency power to high frequency power ratio; Δ index, index during challenge – index at rest.

**TABLE 4:** HRV indices as predictors of cognitive change in subjects with MCI at baseline (*n* = 34).

	Unadjusted model^a^			Adjusted model^b^		
	β	*P*-value	*Q*-value^c^	β	*P*-value	*Q*-value^c^
**Active standing**						
Δ LFn (n.u)^†¶^	−0.531	**0.001**	**0.003**	−0.528	**0.019**	**0.034**
Δ LF/HF^†¶^	−0.639	**< 0.001**	**< 0.001**	−0.643	**0.001**	**0.003**
**Paced breathing**						
Δ LFn (n.u)^‡^	−0.658	**< 0.001**	**< 0.001**	−0.716	**< 0.001**	**< 0.001**
Δ LF/HF^‡^	−0.620	**< 0.001**	**< 0.001**	−0.935	**< 0.001**	**< 0.001**

^a^Simple linear regression with the Δ HRV index as independent variable and the annual change in the cognitive Z-score as dependent variable, ^b^Multiple linear regression adjusted for age, sex, education, physical activity, morbidity (additive index), resting HRV and baseline cognitive Z-score, ^c^P-value corrected for multiple testing by means of the Benjamini–Hochberg procedure with a 5% False Discovery Rate (FDR), ^†^Dependent variable: annual change in episodic memory Z-score (model 1), ^‡^Dependent variable: annual change in executive functioning Z-score (model 2), ^¶^One outlier removed from the analyses. Significant results are shown in bold typeface. MCI, Mild Cognitive Impairment; β, standardized regression coefficient; LFn, normalized low frequency power; n.u, normalized units; LF/HF, low frequency power to high frequency power ratio; Δ index, index during challenge – index at rest.

When the regression analyses were repeated with other Δ HRV indices (TP, LF, HF and time-domain) and with all resting HRV indices (frequency- and time-domain), none were meaningful predictors of cognitive change (with rare and scattered significances not surviving adjustment and/or correction for multiple testing, see Supplementary Tables 6–9).

For reference, the actual values of the HRV indices at rest, during the challenge and their change from rest to challenge are reported in Supplementary Table 10 for LFn and LF/HF and in Supplementary Table 11 for all other indices.

During paced breathing all subjects breathed at the target respiratory rate of 12 breaths/min, as indicated by the center frequency of the HF peak on PSA (0.2 Hz). Also, the respiratory rate was not significantly different between the two groups, both at rest [14.0 (2.5) NC versus 14.7 (2.9) MCI, *t*-test: *p* = 0.276] and during active standing [14.8 (2.7) NC versus 15.6 (3.2) MCI, *t*-test: *p* = 0.290], thus excluding any undue influence of respiratory rate on HRV.

The HR was slightly higher in the MCI group both at rest [65.5 (8.8) NC versus 70.5 (10.0) MCI, *t*-test: *p* = 0.029], during active standing [70.1 (10.2) NC versus 75.3 (10.2) MCI, Mann–Whitney’s *U*-test: *p* = 0.062] and during paced breathing [58.1 (7.0) NC versus 62.0 (8.1) MCI, *t*-test: *p* = 0.033]. Although this is likely to be a spurious finding in the context of multiple testing, and LFn and LF/HF are not affected by HR (Zaza and Lombardi, 2001), we rerun the analyses adjusting for HR (at rest, standing and paced breathing for the resting, Δ standing and Δ paced breathing HRV indices respectively) and found unchanged results (data not shown).

The characteristics of the three cognitive groups at follow-up (NC, MCI and dementia) are shown in Supplementary Tables 12, 13. At baseline, the group who went on to develop dementia was older, had a lower MMSE score and engaged in less physical activity. At follow-up, cognitively impaired subjects unsurprisingly exhibited worse neuropsychological performance than cognitively normal ones. Also, there were no significant between-group differences in the BADL score while the IADL and MMSE scores were lower in subjects with dementia.

## Discussion

The aim of our study was to determine whether the baseline response to a sympathetic versus parasympathetic challenge would differentially predict decline in specific cognitive domains over the follow-up in subjects diagnosed with NC and MCI at baseline. In particular, we hypothesized: (1) that the response to a sympathetic challenge would negatively predict decline in episodic memory, and (2) that the response to a parasympathetic challenge would negatively predict decline in executive functioning. While the second hypothesis was confirmed, the first one was not. In particular, significant findings were confined to the MCI group. The characteristics of the study population were consistent with the relevant literature, the study setting as well as with the exclusion and diagnostic criteria used, and are discussed in the Supplementary Discussion.

### Response to a parasympathetic challenge

The observation that an increased autonomic response to a parasympathetic challenge predicted a slower decline in executive functioning generally aligns with a large body of cross-sectional studies reporting an association between parasympathetic HRV indices and executive functioning (e.g., Williams et al., 2016, 2019; Forte et al., 2019a). More specifically, it conforms well to findings from the sparse longitudinal studies available in the literature. In particular, the standard deviation of the normal to normal intervals (SDNN), which is largely dependent on the parasympathetic nervous system when recorded under resting conditions (Malik et al., 1996), has been shown to have an inverse relationship with decline in the Letter-Digit Coding Test over a 3-year follow-up (Mahinrad et al., 2016) as well as a direct relationship with performance on the Stroop Test 5 years later (Zeki Al Hazzouri et al., 2017) and the Digit-Symbol Coding Test 10 years later (Schaich et al., 2020). Also, a greater parasympathetic responsivity to cognitive tasks, in terms of HF recovery and reactivity, has been demonstrated to predict an attenuated decline in executive functioning (and, to a lesser degree, in episodic memory) across a 9-year period (Knight et al., 2020). Although this latter study was unable to capture an association between the HF reactivity to an orthostatic challenge and changes in cognitive functioning, two points should be noted. First, the orthostatic stress was less intense since it involved standing from a sitting rather than a supine position. Second, there was no recovery period following the orthostatic challenge and no “true” baseline since HF reactivity was computed as HF during standing minus HF during the recovery epoch of the cognitive challenges. Given that mental stress produces parasympathetic withdrawal (Castaldo et al., 2015), as does orthostatic stress, such methodological choice is bound to reduce the magnitude of the orthostatic HF response and thus the likelihood of detecting any potential association it may have with cognition. Furthermore, even if Britton et al. (2008) concluded for no association between HRV and cognitive impairment, due to the lack of consistency of the observed associations across cognitive domains, they did identify a relationship between lower SDNN and HF and a greater 5-year decrease in the Mill Hill Test, which can be considered a measure of executive functioning (Raven, 1983). Lastly, a very recent study by Costa et al. (2021) has found that greater decline in executive functioning tests like the Digit-Symbol Coding and the Digit Span Forwards and Backwards over 6 years was predicted by greater heart rate fragmentation (HRF). This is a novel HRV metric whose physiological underpinnings are still unresolved, but are likely reflective of a degradation of the parasympathetic nervous system (Costa et al., 2017). The authors do not find an association between cognitive decline and traditional parasympathetic HRV indices [SDNN, the root mean square of successive differences of the normal to normal intervals (RMSSD) and HF], but the use of ECG recordings from in-home overnight polysomnography from an ancillary sleep study is an approach that, albeit convenient, carries some limitations. The presence of sleep-disordered breathing, highly prevalent in the study population (Chen et al., 2015), can have a confounding effect on HRV analysis both because it actually alters autonomic control and because abnormal breathing patterns distort the interpretation of the power spectrum (Tobaldini et al., 2013). The same would be true of leg movements during sleep, which are linked to autonomic activation as well as a potential source of motion artifacts (Guggisberg et al., 2007; Tobaldini et al., 2013). Moreover, despite the fact that the stationarity requirements of spectral analysis warrant the averaging of values from 5-min windows over the entire 12-h period, such averaging obscures detailed information on autonomic modulation (Malik et al., 1996).

### Response to a sympathetic challenge

The finding that an increased autonomic response to a sympathetic challenge predicted a faster decline in episodic memory was contrary to our expectations. Indeed, sparse cross-sectional studies have shown that better episodic memory is associated with enhanced sympathetic activation, as indexed by increased sympathetic HRV or decreased parasympathetic HRV. In fact, LF/HF has been found to positively correlate with the episodic memory subdomain of the Montreal Cognitive Assessment (MOCA) score in older adults (Frewen et al., 2013), orthostatic Δ LFn and Δ LF/HF have been reported to positively correlate with the Prose Delayed Recall Z-score in subjects with amnestic MCI (Nicolini et al., 2020), and a stress-induced increase in SDNN has been noticed to positively correlate with the California Verbal Learning Task in healthy individuals of different ages (Hilgarter et al., 2021). Also, resting TP, which is mostly parasympathetically-mediated (Malik et al., 1996), and HF, which is a parasympathetic index, have been found to negatively correlate with Word Recall and Word/Picture Recognition in late-life depression (Vasudev et al., 2012) and with the Seoul Verbal Learning Test in subjects with MCI/dementia (Kim et al., 2018). Along the same lines, Dalise et al. (2020) have described a positive adjusted association between LF/HF and the MMSE score in geriatric outpatients representative of a real-life setting, and it is recognized that, especially at older ages, the MMSE primarily captures episodic memory (Soubelet and Salthouse, 2011; Dichgans and Leys, 2017).

There could be different potential explanations for the association between the sympathetic response and the change in episodic memory being in the opposite direction than anticipated.

First, it could be a paradoxical effect stemming from survivor bias. In fact, low HRV has been demonstrated to increase mortality in older adults (Nicolini et al., 2012) and individuals with higher HRV, by living longer, could have a greater opportunity to develop cognitive impairment. However, this interpretation seems unlikely since, in our study, loss to follow-up was small (11%) and well below the 20–40% threshold recommended for cohort studies (Ramirez-Santana, 2018). Also, sympathetic Δ HRV indices were not significantly different between the mortality and no-mortality groups (see Supplementary Table 14).

Second, it could reflect a regression to the mean phenomenon by which, on repeated neuropsychological testing, more extreme scores at baseline tend to converge toward the mean at follow-up, so that there is a negative association between baseline and change cognitive scores. If this is coupled with a positive association between baseline sympathetic Δ HRV indices and baseline episodic memory, the former would then have a negative longitudinal relationship with change in episodic memory, i.e., greater baseline sympathetic Δ HRV indices would be linked to greater episodic memory decline at follow-up. Nonetheless, this does not appear to be plausible because there was no significant cross-sectional association between the Δ HRV indices and cognitive tests at baseline (see Supplementary Tables 15, 16 and the Supplementary Discussion) and because, in any case, regression to the mean was controlled for by adjusting the regression analyses for baseline cognitive scores (Yu and Chen, 2015).

Therefore, it is reasonable to assume that our results do not represent a statistical artifact, but, rather, have a true pathophysiological basis. Support for this view comes from a wealth of functional neuroimaging studies that have primarily focused on individuals with MCI (O’Brien et al., 2010; Sperling et al., 2010; Huijbers et al., 2015), mostly of the amnestic type, but have also spanned the cognitive continuum to include individuals with mild AD dementia (Alsop et al., 2008; Sperling et al., 2010) as well as cognitively normal older adults at genetic risk (Sperling et al., 2010; Rao et al., 2015) or not (Leal et al., 2017) for AD. They have revealed that in these subjects, relatively to controls, there is mostly a hyperactivity of the hippocampal/parahippocampal region (Alsop et al., 2008; O’Brien et al., 2010; Sperling et al., 2010; Huijbers et al., 2015; Rao et al., 2015) and, additionally, that hyperactivity is a longitudinal predictor of greater cognitive decline (O’Brien et al., 2010; Sperling et al., 2010; Huijbers et al., 2015; Rao et al., 2015; Leal et al., 2017), especially in episodic memory (O’Brien et al., 2010; Rao et al., 2015; Leal et al., 2017). These data fit in nicely with the cognitive reserve theory (Stern, 2012) according to which the brain can compensate for damage by engaging neural networks. Within this context, hyperactivity is viewed as a compensatory attempt to maintain cognitive performance in the face of cerebral pathology (Sperling et al., 2010; Stern, 2012). Likewise, hyperactivity is a harbinger of cognitive decline because it signifies that the system is working closer to its maximum capacity, i.e., closer to the threshold beyond which reserve is depleted and compensation is no longer possible (Sperling et al., 2010; Stern, 2012).

Somewhat more recently, similar evidence has emerged for the insula. Hyperactivity of the insular cortex has been demonstrated in subjects with MCI (Scarmeas et al., 2004; Alexopoulos et al., 2012; Alfini et al., 2019), mild AD dementia (Scarmeas et al., 2004) and cognitively normal older adults at genetic risk of AD (Thambisetty et al., 2010; Lin et al., 2017). Also, in subjects with MCI, insular hyperactivity has been associated with greater cognitive decline at follow-up (Devanand et al., 2006).

Although there is little functional (and even structural) neuroimaging work targeting the locus coeruleus, due to the technical challenges of imaging a small brainstem nucleus (Mather, 2021), other lines of research converge toward the notion that it, too, may exhibit an inverse U-shaped activation curve in AD (e.g., Ross et al., 2015). Thus, noradrenergic transmission would be aberrantly increased in the earlier stages and then decline later on (e.g., Ross et al., 2015).

On the whole, it can be conjectured that a greater HRV response to a sympathetic challenge at baseline could be indexing an increased activity of those cerebral regions that generate sympathetic outflow (i.e., the hippocampus/parahippocampus, insula and locus coeruleus) and that such hyperactivation may be signaling the impeding breakdown of compensatory mechanisms. Accordingly, as brain damage progresses, a point will be reached during the follow-up when compensation can no longer occur and there is a rapid deterioration of cognitive performance in the domains underpinned by these brain structures, i.e., episodic memory. It should be noted that the negative (and not positive) longitudinal association between the response to a parasympathetic challenge and executive decline would appear to imply that compensation is not operating here, and a potential reason for this is given the Supplementary Discussion.

### Strength and limitations

The study has strengths and limitations. The study strengths include its novelty as well as the performance of a detailed and *ad hoc* neuropsychological, autonomic and clinical assessment including extensive neuropsychological testing, the use of two physical maneuvers to differentially challenge the two branches of the ANS and the collection of information on several potential confounders.

A number of limitations must also be acknowledged. Since HRV was not measured at follow-up it was not possible to track the trajectory of autonomic function over time. This would have enabled us to more thoroughly examine the relationship between HRV and cognition by determining how change in HRV associates with change in cognition (Elias and Torres, 2017), an issue which still remains unexplored. Thus, future longitudinal research should include concurrent HRV and cognitive measures.

The sample size was relatively small. Although an *a posteriori* power analysis with a large expected effect size revealed an almost 100% power for multiple linear regression (see “Statistical analysis” section), only in the MCI group the observed effect size was large according to Acock (2014) (standardized beta coefficient > 0.5). Therefore, the study may have been underpowered to detect smaller effect sizes in the NC subjects and our findings will need to be replicated in larger cohorts.

Only the HRV indices that were the main focus of the study (i.e., Δ LFn and Δ LF/HF) were found to be significant predictors of cognitive change. No meaningful results were observed for other Δ HRV indices (time- and frequency-domain) or for all resting HRV indices. This was probably the case because it is generally recognized that, in short-term ECG recordings performed in controlled experimental conditions, there is a greater sensitivity of the spectral and Δ indices (Malik and Camm, 1990; Montano et al., 2009; Malik et al., 2019). The fact that this was not true of other Δ spectral indices (i.e., Δ HF, Δ TP and Δ LF) is likely due to their statistical properties and/or physiological significance. Although HF is an index of parasympathetic modulation it is also a highly dispersed variable (Nunan et al., 2010). TP and LF are indices of joint sympathetic and parasympathetic modulation, which implies that, since sympathetic activity is related to episodic memory and parasympathetic activity is related to executive functioning, a “mixed” measure is less able to capture these domain-specific associations, because their strength is diluted by the presence of a second autonomic component unrelated to the specific cognitive domain. It cannot be excluded that with a larger sample size and/or PSA techniques based on the Welch method, which reduces HRV variance (Stoica and Moses, 2004), our findings could have been different. Also, it should be noted that, even if transformed HRV indices (i.e., LFn and LF/HF) have received ample support in the literature (e.g., Malliani et al., 1998; Montano et al., 2009; Malik et al., 2019) and have been extensively employed in HRV studies across topics (e.g., Niu et al., 2018; Dalise et al., 2020; Sun et al., 2021), they have also been questioned (e.g., Heathers, 2014). Future works should address other measures of sympathetic activity such as plasma adrenaline/noradrenaline levels (Goldstein et al., 2011) and the pre-ejection period (Wiley et al., 2021).

The nature of the sample (i.e., outpatients referred to a Geriatric Unit) can limit the generalizability of our study to the older community-dwelling population at large, and the exclusion of individuals with dementia at baseline implies that HRV was not investigated as a predictor of cognitive decline in this subset of older adults.

Lastly, although the role of HRV as indicator of brain activity is grounded in much literature (see “Introduction,” Supplementary Introduction, and “Discussion” sections), the study lacked functional neuroimaging which could have provided deeper insight into the complexity of the relationship between HRV, cognition and the activity of specific CAN regions.

## Conclusion

Our study is the first to show that the HRV response to a physical challenge predicts cognitive decline in a clinically relevant population of older adults, i.e., subjects with MCI. In particular, a differential effect was observed for the two branches of the ANS and for the episodic memory versus executive functioning domain. These findings contribute to the sparse existing literature on the prospective association between autonomic and cognitive processes, and support the emerging role of HRV as a biomarker for cognitive impairment and, potentially, as a target for intervention. Our results contribute to the understanding of the heart-brain connection, but will require confirmation from future research on larger cohorts with concurrent HRV and cognitive measurements as well as functional neuroimaging data.

## Data availability statement

The raw data supporting the conclusions of this article will be made available by the authors, without undue reservation.

## Ethics statement

The studies involving human participants were reviewed and approved by Fondazione IRCCS Ca’ Granda Ospedale Maggiore Policlinico. The patients/participants provided their written informed consent to participate in this study.

## Author contributions

PN conceived and designed the study, acquired and interpreted the data, and drafted and critically revised the manuscript. CA, SI, ET, and PR acquired and interpreted the data. DM and TL interpreted the data. MV interpreted and critically revised the data. All authors contributed to the manuscript and approved the submitted version.
