# Supplementary material for: Autonomic function predicts cognitive decline in mild cognitive impairment: Evidence from power spectral analysis of heart rate variability in a longitudinal study
Source: Front Aging Neurosci. 2022 Sep 15;14:886023. doi: 10.3389/fnagi.2022.886023 (PMC9520613; doi:10.3389/fnagi.2022.886023)
Supplement: Supplementary file 1 [file Data_Sheet_1.docx]

**Supplementary Material**

**1. Supplementary Introduction**

- 1. **Limitations of longitudinal studies on HRV and cognitive functioning**

**1.1.1 ECG recordings**

Half of the studies use routine 10-second ECG recordings (Mahinrad et al., 2016; Zeki Al Hazzouri et al., 2017; Schaich et al., 2020) and in all studies except one (Knight et al., 2020) the recording is performed in resting conditions. The reliability of HRV measures derived from ultra-short-term recordings, i.e. below the 5-minute standard recommended by the Task Force of the European Society of Cardiology and the North American Society for Pacing and Electrophysiology (Malik et al., 1996), is questionable, especially for the time-domain indices and for durations below 1 to 2 minutes (Malik et al., 1996; Laborde et al., 2017; Shaffer and Ginsberg, 2017). In addition, the assessment of HRV is less sensitive in detecting autonomic dysfunction when it is performed in resting conditions rather than in response to provocative tests that challenge the ANS (Montano et al., 2009; Malik et al., 2019).

**1.1.2 Cognitive tests**

All studies rely on limited cognitive batteries and not on extensive neuropsychological testing. The number of cognitive tests ranges from 3 (Mahinrad et al., 2016; Zeki Al Hazzouri et al., 2017; Schaich et al., 2020; Costa et al., 2021) to 6 (Knight et al., 2020), with Britton et al. (2008) using 5 tests. Also, the cognitive tests are mainly restricted to executive functioning. In fact, the cognitive batteries tap from 2 (Mahinrad et al., 2016; Zeki Al Hazzouri et al., 2017; Schaich et al., 2020; Costa et al., 2021) to 5 (Knight et al., 2020) executive domains, but only one component of episodic memory, either verbal (Britton et al., 2008; Zeki Al Hazzouri et al., 2017; Knight et al., 2020) or visual (Mahinrad et al., 2016).

**1.1.3** **Lack of cognitive data at baseline**

While all studies explore the relationship between baseline HRV and cognition, only four do so in a more comprehensive manner by taking cognitive change from baseline to follow-up as outcome measure (Britton et al., 2008; Mahinrad et al., 2016; Knight et al., 2020; Costa et al., 2021). The other two studies lack cognitive data at baseline and can merely evaluate the link between baseline HRV and cognitive performance 5 (Zeki Al Hazzouri et al., 2017) or 10 (Schaich et al., 2020) years later.

**1.1.4 Paucity of findings on episodic memory**

Although the studies provide generally consistent evidence of an association between parasympathetic HRV indices and executive functioning, findings concerning episodic memory are much more scant. This lack of information is problematic because episodic memory is a critical dimension of cognitive function (Staresina and Wimber, 2019) which is preferentially affected by both normal (Dahan et al., 2020) and pathological (i.e. AD-related) (Bastin and Salmon, 2014) cognitive aging. Yet, episodic memory is either not tested (Schaich et al., 2020; Costa et al., 2021) or reported to be unrelated to HRV (Britton et al., 2008; Mahinrad et al., 2016; Zeki Al Hazzouri et al., 2017) or found to be less strongly associated with HRV than executive functioning (Knight et al., 2020). There may be several methodological reasons for this, including the HRV and cognitive measures employed as well as the nature of the study populations. The HRV metrics mostly reflect parasympathetic modulation, which has been shown to be primarily associated with executive functioning (e.g. Williams et al., 2016; Forte et al., 2019a; Williams et al., 2019). The cognitive batteries assess only one aspect of episodic memory (verbal or visual) while both have been recognized to be vulnerable to AD (e.g. Bastin and Salmon, 2014). The study populations are composed of middle-aged/ middle-aged to older adults (Britton et al., 2008; Zeki Al Hazzouri et al., 2017; Knight et al., 2020; Schaich et al., 2020; Costa et al., 2021) or specifically selected older subjects (Mahinrad et al., 2016). In middle age, greater deficits have been demonstrated in executive functioning than in episodic memory (Singh-Manoux et al., 2012; Hughes et al., 2018). In later life, episodic memory is more commonly impaired (Dahan et al., 2020), but Mahinrad et al. (2016) analyzed data from the PROSPER (Prospective Study of Pravastatin in the Elderly at Risk) trial which recruited older subjects with a history of, or risk factors for, cardiovascular disease and a MMSE score ≥ 24. Since cardiovascular risk/disease is linked to executive dysfunction (Rostamian et al., 2015; Veldsman et al., 2020) and the MMSE is memory-focused (Soubelet and Salthouse 2011; Dichgans and Leys, 2017), the study participants would be expected to exhibit greater executive than episodic memory impairment and indeed, based on neuropsychological test normative data (Barbarotto et al., 1998; Van der Elst et al., 2005; Van der Elst et al., 2006), this seems to be the case (Mahinrad et al., 2016; Houx et al., 2002). Thus, taken together, the demographic and clinical features of the samples, along with the choice of cognitive tests and HRV indices, suggest that the association between HRV and episodic memory may have been underestimated.

**1.2 Literature review on the site-specific role of the CAN in cognition and autonomic control**

**1.2.1 Hippocampus/parahippocampus**

**1.2.1.1 Cognition**

The notion of the hippocampal/parahippocampal region as a critical hub for episodic memory is widely accepted and has been established by a plethora of work including lesion studies (e.g. Dickerson and Eichenbaum, 2010) and electrophysiological recordings (e.g. Dickerson and Eichenbaum 2010; Staresina and Wimber, 2019) in animals and humans, as well as functional neuroimaging techniques in humans (e.g. Nyberg, 2017; Staresina and Wimber, 2019).

**1.2.1.2 Autonomic control**

Although the hippocampus/parahippocampus has received less attention in terms of autonomic regulation, there is a growing consensus, despite some controversy (e.g. Ruit and Neafsey, 1988), that it is a site of sympathetic output. In animal models, chemical (Lathers et al., 1993; Khookhor and Umegaki, 2013) and electrical (Aitake et al., 2011) stimulation of the hippocampus increased HR (Lathers et al., 1993; Aitake et al., 2011), BP (Lathers et al., 1993) and LF/HF (Khookhor and Umegaki, 2013). The stress response in rodents has been found to parallely increase hippocampal theta power and LF/HF (Aitake et al., 2011), and to be attenuated by the inhibition of hippocampal glutamatergic transmission (Moraes-Neto et al., 2014). In humans, functional neuroimaging has documented activation of the hippocampus during tasks eliciting sympathoexcitation (Kimmerly, 2017), a positive correlation between task-induced hippocampal/parahippocampal activation and skin conductance activity (a marker of sympathetic tone) in adolescents (Weissman et al., 2018) and young adults (Nagai et al., 2004), as well as an attenuated hippocampal activation in response to a stress task in blunted cardiac reactors (Ginty et al., 2013). Also, resting HF has been reported to be negatively correlated with the perfusion (Allen et al., 2015) and grey matter volume (Wei et al., 2018) of the hippocampus and parahippocampal gyrus (Allen et al., 2015; Wei et al., 2018) on Magnetic Resonance Imaging (MRI). A recent functional MRI (fMRI) study at rest (Valenza et al., 2020) has identified a negative association between brain activity in the hippocampus/parahippocampal gyrus and a complexity HRV index that decreases with sympathetic activation (Valenza et al., 2012).

**1.2.2 Insula**

**1.2.2.1 Cognition**

The insula has also been linked to episodic memory by a number of neuroimaging studies in humans. On functional neuroimaging, insular activation has been observed during episodic memory tasks in healthy adults (Spaniol et al., 2009; Kurth et al., 2010). On structural and functional neuroimaging, positive correlations have been highlighted between scores on episodic memory tests and the grey matter volume (Chen J. et al., 2015), glucose metabolism (Seo and Choo, 2016) and functional connectivity (Xie et al., 2012) of the insular region in subjects with amnestic MCI.

**1.2.2.2 Autonomic control**

The insular cortex has also been consistently recognized as a key brain region for the production of sympathetic outflow. In rodents, electrical and chemical stimulation of the insula increased HR and BP (Ruggiero et al., 1987). In humans, functional neuroimaging studies have revealed that, during tasks that stimulate the sympathetic nervous system, there occurs an activation of the insula that correlates with increases in muscle sympathetic nerve activity (Kimmerly, 2017). Along the same lines, greater stressor-evoked cardiovascular reactivity has been found to be associated with greater activation of the insula (Gianaros et al., 2005; Ginty et al., 2013). Moreover, HF at rest has been reported to have a negative relationship with the grey matter volume (Wei et al., 2018) and perfusion (Allen et al., 2015; Valenza et al., 2019) of the insula on MRI.

**1.2.3 Locus coeruleus**

The locus coeruleus, although somewhat neglected in previous literature, is currently gaining interest as the first brain region affected by the neuropathological changes of AD (Theofilas et al., 2107), as well as for its noradrenaline-mediated effects on cognitive (Mather and Harley, 2016) and autonomic (Mather et al., 2017) function.

**1.2.3.1 Cognition**

Rodent behavioural studies have shown that the optogenetic activation of the locus coeruleus potentiated learning in an episodic memory task (McNamara and Dupret, 2017). Likewise, in older adults the integrity of the locus coeruleus on neuromelanin-sensitive MRI scans has been found to be positively associated with episodic memory performance (Hämmerer et al., 2018; Dahl et al., 2019).

**1.2.3.2 Autonomic control**

The locus coeruleus is acknowledged to be a crucial node of the sympathetic nervous system that also suppresses parasympathetic activity (Samuels and Szabadi, 2008). Accordingly, in healthy subjects across the age span, a link has been noted between lower HF and increased locus coeruleus activity on MRI. In younger and older adults, greater locus coeruleus MRI contrast on neuromelanin-sensitive scans was negatively associated with HF during fear conditioning and spatial detection tasks (Mather et al., 2017). In young adults, pain stimulation reduced HF and increased the blood oxygen level-dependent signal from the locus coeruleus on ultrahigh-field fMRI (Sclocco et al., 2016).

**1.2.4 Prefrontal cortex**

**1.2.4.1 Cognition**

A wealth of data from lesion studies in animals (e.g Chudasama, 2011; Kesner and Churchwell, 2011) and humans (e.g. Alvarez and Emory, 2006) as well as from neuroimaging studies in humans (e.g. Alvarez and Emory, 2006; Yuan and Raz, 2014) have indicated that executive functions have their neural basis in the prefrontal cortex.

**1.2.4.2 Autonomic control**

Research by Thayer and others (e.g. Thayer et al., 2009; Mather and Thayer, 2018) has led to the elaboration of a neurovisceral integration model by which prefrontal cortical activity is indexed by parasympathetic HRV. Indeed, a substantial body of evidence from animal and human studies lends support to such conceptual framework. In rats, electrical (Sun, 1992; Owens and Verberne, 2001) and chemical (Owens and Verberne, 2001) stimulation of the prefrontal cortex produced a depressed cardiovascular response. In humans, functional neuroimaging has found that, during sympathoexcitatory tasks, there is a deactivation of the prefrontal cortex which correlates with the increase in muscle sympathetic nerve activity (Kimmerly, 2017). Moreover, structural and functional neuroimaging have linked parasympathetic HRV to the thickness, blood flow and functional connectivity of the human prefrontal cortex (Wood et al., 2017; Mather and Thayer, 2018; Schumann et al., 2021). Similarly, prefrontal activation on electroencephalography has been reported to be associated with increased HF (Patron et al., 2019). Lastly, stimulation studies in humans have documented that prefrontal transcranial direct stimulation increases HF (Nikolin et al., 2017) and that transcutaneous auricular vagus nerve stimulation increases both parasympathetic HRV and the electroencephalographic activity of the frontal cortex (Machetanz et al., 2021).

**2. Supplementary Materials and Methods**

**Diagnosis of dementia**

All-cause dementia was diagnosed according to the NIA-AA core clinical criteria which require impairment in at least two cognitive domains that significantly interferes with the activities of daily living (McKhann et al., 2011). Thus, the diagnosis relied on a combination of neuropsychological testing and functional status assessment by means of the BADL and IADL scales. The same cut-off as for MCI was used to define a pathological test score (i.e. below the 10th percentile of the normative score distribution), consistently with the notion that the key differential feature between MCI and dementia is the functional impact of the cognitive deficits (McKhann et al., 2011; Gorelick et al., 2011; Petersen et al., 2018; Jack et al., 2018). Nonetheless, subjects with dementia were found to score lower than those with MCI on many cognitive tests (see Supplementary Table 13) because of the expected correlation between functional and cognitive impairment.

As to the specific diagnosis of AD dementia or VAD, it was based on the NIA-AA (McKhann et al., 2011) and AHA/ASA (Gorelick et al., 2011) criteria respectively, and it was assisted by a number of diagnostic tools including neuropsychological assessment, routine blood tests, clinical assessment, and biomarkers (neuroimaging and CSF). Unlike the rest of this diagnostic work-up, which was standard for all participants, the choice of biomarkers was made by the geriatrician on a case-by-case basis as appropriate.

The neuropsychological assessment (see Materials and Methods in the main text) can identify characteristic patterns of cognitive deficits: an amnestic syndrome of the hippocampal type with prominent deficits in episodic memory in the case of AD dementia (McKhann et al., 2011; Dubois et al., 2014) and a prefrontal dysexecutive syndrome in the case of subcortical VAD, the most common form of VAD (Smith, 2017; Gorelick et al., 2011). Routine blood tests included vitamin B12, folic acid and thyroid function in order to rule out treatable causes of dementia (Knopman et al., 2010).

The clinical assessment comprised clinical history-taking and a neurological examination to investigate non-AD non-VAD neurological conditions as well as non-neurological medical conditions, and to collect potential clinical evidence of VAD (e.g. history of stroke and focal neurological signs in cortical VAD, more subtle neurological signs such as gait impairment in subcortical VAD) (Smith, 2017; Mc Khann et al., 2011; Gorelick et al., 2011). The biomarkers encompassed structural and functional neuroimaging as well as CSF analysis. Structural imaging by CT and/or MRI can visualize significant hippocampal atrophy and significant cerebrovascular burden in AD dementia and VAD respectively (McKhann et al., 2011; Gorelick et al., 2011). Fluorodeoxyglucose-Positron Emission Tomography (FDG-PET) shows decreased FDG uptake in the temporo-parietal cortex in AD dementia (McKhann et al., 2011). The CSF profile of AD dementia is marked by low levels of amyloid-beta (Aβ _1-42_) and high levels of tau (total and/or phosphorylated) (McKhann et al., 2011). Overall, in the 12 subjects diagnosed with dementia, brain MRI was the most frequently requested exam (n = 10), followed by brain CT (n = 3) and lumbar puncture (n = 3), and by FDG-PET (n = 2).

**3.1 Characteristics of the study population**

The mortality rate of the sample was about 3% per year, which is lower than the national data for the Italian population within the same age range (seventh to ninth decade: 4% to 25%) (Istat, 2021), consistently with the exclusion of a number of severe comorbidities.

The rates of conversion from NC to MCI and from MCI to dementia were around 11% and 12% per year, respectively. Although there is a large variability in conversion rates across studies due to several methodological reasons such as recruitment source, cognitive tests used, operationalization of the MCI criteria and length of follow-up (Roberts and Knopman, 2013), the values we found appear to be somewhat intermediate between those from clinic- and community-based cohorts. In fact, rates of conversion have been shown to be, in community versus clinic settings, 5% versus 30% for NC to MCI (Chen et al., 2017), and 3% versus 13-24% for MCI to dementia (Maioli et al., 2007; Farias et al., 2009). This seems to be in keeping with the fact that we enrolled subjects from a clinical context, i.e. at higher risk of progression, but then selected the ones with better health, i.e. devoid of most vascular risk factors and diseases, all of which are known to accelerate cognitive decline (Roberts and Knopman, 2013).

Also, the distribution of dementia types (75% AD dementia, 25% VAD) was in line with the recognized prevalence of different forms of dementia (Alzheimer’s Society, 2020).

Only one individual reverted from MCI to NC, yielding a reversion rate of about 1% per year, which agrees with the figure reported by others (Gallassi et al., 2010). Likewise, only one NC subject developed dementia without an interim diagnosis of MCI. The rate of direct progression from NC to dementia was therefore around 1% per year, which is very close to the 0.6% observed by Michaud et al. (2017).

Progression to dementia was more likely with increasing age as well as with lower MMSE and physical activity at baseline (see Supplementary Table 12). These results are consonant with the literature on predictors of cognitive decline (e.g. Roberts and Knopman, 2013).

Just as unsurprisingly, neuropsychological performance at follow-up was worse in cognitively impaired individuals, i.e. in subjects diagnosed with MCI and dementia, than in cognitively normal ones (see Supplementary Table 13). Then, based on the specific test, significant differences in test scores were observed either across the cognitive spectrum or between NC and cognitively impaired (MCI/dementia) subjects or between NC/MCI and dementia subjects (see Supplementary Table 13).

The MMSE had a lower score at follow-up in participants with dementia, but displayed no statistically significant difference between subjects with NC and MCI (see Supplementary Table 13), in accordance with its known poor sensitivity for MCI (Lonie et al., 2009).

As far as functional status at follow-up was concerned, the BADL score was not significantly different between groups, while the IADL score was lower in subjects with dementia than in subjects with NC or MCI (see Supplementary Table 13). These findings are attuned to the established notion that, over the course of cognitive decline, the ability to perform daily activities becomes first impaired for the more cognitively complex tasks (e.g. Marshall et al., 2012), so that in mild dementia BADL are preserved unlike IADL. Indeed, when considering the severity of dementia (National Institute for Clinical Excellence (NICE), 2011), in our study sample most subjects with dementia had mild dementia (n = 10, 83%), with a MMSE score between 21 and 26, while only two had moderate dementia, with a MMSE score between 10 and 20.

**3.2 Additional considerations**

Some points regarding the relationship between HRV and cognition deserve further mention.

First, the negative (and not positive) longitudinal association between the response to a parasympathetic challenge and executive decline would appear to imply that compensation is not operating here. Since compensation increases with increasing damage (e.g. Sperling et al., 2010; Gold et al., 2017) it may be speculated that there is a level of damage before which no compensation takes place. Indeed, in subjects with MCI, compensation by increased prefrontal cortex connectivity has been reported to occur in those with a higher vascular risk factor burden (Chen et al., 2018). Given that the study exclusion criteria comprised a number of vascular risk factors and diseases that are linked to frontal lobe damage (Smith, 2017), it is likely that in our sample there was less vascular damage to the frontal lobe than there were neurodegenerative changes in the hippocampus/parahippocampus, insula and locus coeruleus. Although the original work was not designed to include neuroimaging data, in a subsequent extension of the study (Nicolini et al., 2020) we also evaluated brain computed tomography scans, by means of semiquantitative scales, in all MCI subjects and in the NC subjects for whom they had been prescribed. Notwithstanding the possible shortcomings of this approach (e.g. structural and not functional imaging, no volumetric assessment, partial data availability), if we carry out a sensitivity analysis on the subgroup belonging to the current study, we find that, as presumed, total cerebrovascular burden was not significantly different between the NC and MCI subjects, but hippocampal atrophy was greater in the MCI subjects (see Supplementary Table 17).

Second, despite the fact that HRV and cognition are linked by a common neural substrate (see Supplementary Introduction), the longitudinal associations found between the ∆ HRV indices and specific cognitive domains were not also present cross-sectionally. Some inconsistency between cross-sectional and longitudinal associations has also been noted by other studies on HRV and cognition (e.g. Mahinrad et al., 2016; Schaich et al., 2020; Costa et al., 2021). We believe a likely explanation is that, although both HRV and neuropsychological performance are regarded as surrogate measures of brain activity, HRV has a potential for increased sensitivity since it is a more “upstream” marker of cerebral function (Tan et al., 2014) and does not suffer from a number of deficiencies inherent in neuropsychological testing, including population-based norming (Howieson, 2019). Thus, early disruption of brain activity would be indexed by HRV but not by cognition, producing a lack of association between the two. With the progression of brain damage over time, cognitive dysfunction would then emerge as an indicator of reduced brain activity, being greatest for individuals with greater disrupted brain activity at baseline, resulting in an association between HRV at baseline and cognitive decline across the follow-up. In other words, the occurrence of a time-lag between the respective abilities of HRV and cognition to mirror brain function could preclude their cross-sectional association and lead instead to a prospective relationship in which altered HRV heralds cognitive change. An alternative explanation could be that there is a direct causal link between autonomic and cognitive dysfunction, with the former causing the latter after an appropriate causal interval, meaning that a longitudinal study design would be the best suited to highlight such association. This prospective effect could be for instance mediated by BP dysregulation, whether in terms of absolute BP values (Forte et al., 2019b) or BP variability (BPV) (Jia et al., 2021). It should, however, be emphasized that these hypotheses remain speculative and will need to be elucidated by further research since our study was not intended to quantify brain damage or explore BP regulation.

**Supplementary Tables**

**Supplementary Table 1**. Neuropsychological test battery

| Cognitive domain | Neuropsychological test | Reference |
| --- | --- | --- |
| Episodic memory | Prose recall  ROCF-delayed recall | Carlesimo et al., 2002 |
| Executive functions | Bell Test | Vallar et al., 1994 |
|  | Digit Cancellation Test | Spinnler and Tognoni, 1987 |
|  | Digit Span Forwards | Orsini et al., 1987 |
|  | Digit Span Backwards | Monaco et al., 2013 |
|  | Trail-Making Test A | Giovagnoli et al., 1996 |
|  | Trail-Making Test B |  |
|  | Weigl’s Test | Spinnler and Tognoni, 1987 |
|  | Cognitive Estimates-total | Della Sala et al., 2003 |
|  | Cognitive Estimates-bizarre |  |
|  | Raven’s CPM | Spinnler and Tognoni, 1987 |
|  | Letter fluency | Novelli et al., 1986 |
| Language | Category fluency  Picture naming  Token Test | Spinnler and Tognoni, 1987  Laiacona et al., 1993  Spinnler and Tognoni, 1987 |
| Visuospatial skills | ROCF-copy  Copy of geometric figures | Caffarra et al., 2002  Spinnler and Tognoni, 1987 |
| Ideomotor praxis | De Renzi’s Test-right upper limb  De Renzi’s test-left upper limb | De Renzi et al., 1980 |

**Legend**

Abbreviations: ROCF, Rey-Osterrieth Complex Figure; CPM, Colored Progressive Matrices.

**Supplementary Table 2**. HRV indices as predictors of cognitive change in subjects with NC at baseline (n = 37)

|  | Unadjusted model ^a^ | | | Adjusted model ^b^ | | |
| --- | --- | --- | --- | --- | --- | --- |
| Active standing | β | P-value | Q-value ^c^ | β | P-value | Q-value ^c^ |
| ∆ LFn (n.u) ^†^ | 0.166 | 0.327 | 0.476 | 0.208 | 0.218 | 0.348 |
| ∆ LF/HF ^†^ | -0.395 | **0.016** | **0.031** | -0.147 | 0.382 | 0.500 |
| Paced breathing |  |  |  |  |  |  |
| ∆ LFn (n.u) ^‡¶^ | -0.086 | 0.617 | 0.690 | 0.092 | 0.646 | 0.690 |
| ∆ LF/HF ^‡¶^ | -0.143 | 0.406 | 0.500 | 0.070 | 0.783 | 0.783 |

**Legend**

^a^ Simple linear regression with the ∆ HRV index as independent variable and the annual change in the cognitive Z-score as dependent variable, ^b^ Multiple linear regression adjusted for age, sex, education, physical activity, morbidity (Z-score), resting HRV and baseline cognitive Z-score, ^c^ P-value corrected for multiple testing by means of the Benjamini-Hochberg procedure with a 5% False Discovery Rate (FDR), ^†^ Dependent variable: annual change in episodic memory Z-score (model 1), ^‡^ Dependent variable: annual change in executive functioning Z-score (model 2), ^¶^ One outlier removed from the analyses. Significant results are shown in bold typeface. Abbreviations: NC, Normal Cognition; β, standardized regression coefficient; LFn, normalized low frequency power; n.u, normalized units; LF/HF, low frequency power to high frequency power ratio; ∆ index, index during challenge – index at rest.

**Supplementary Table 3**. HRV indices as predictors of cognitive change in subjects with MCI at baseline (n = 34)

|  | Unadjusted model ^a^ | | | Adjusted model ^b^ | | |
| --- | --- | --- | --- | --- | --- | --- |
| Active standing | β | P-value | Q-value ^c^ | β | P-value | Q-value ^c^ |
| ∆ LFn (n.u) ^†¶^ | -0.531 | **0.001** | **0.003** | -0.519 | **0.021** | **0.037** |
| ∆ LF/HF ^†¶^ | -0.639 | **< 0.001** | **< 0.001** | -0.636 | **0.001** | **0.003** |
| Paced breathing |  |  |  |  |  |  |
| ∆ LFn (n.u) ^‡^ | -0.658 | **< 0.001** | **< 0.001** | -0.702 | **< 0.001** | **< 0.001** |
| ∆ LF/HF ^‡^ | -0.620 | **< 0.001** | **< 0.001** | -0.924 | **< 0.001** | **< 0.001** |

**Legend**

^a^ Simple linear regression with the ∆ HRV index as independent variable and the annual change in the cognitive Z-score as dependent variable, ^b^ Multiple linear regression adjusted for age, sex, education, physical activity, morbidity (Z-score), resting HRV and baseline cognitive Z-score, ^c^ P-value corrected for multiple testing by means of the Benjamini-Hochberg procedure with a 5% False Discovery Rate (FDR) , ^†^ Dependent variable: annual change in episodic memory Z-score (model 1), ^‡^ Dependent variable: annual change in executive functioning Z-score (model 2), ^¶^ One outlier removed from the analyses. Significant results are shown in bold typeface. Abbreviations: MCI, Mild Cognitive Impairment; β, standardized regression coefficient; LFn, normalized low frequency power; n.u, normalized units; LF/HF, low frequency power to high frequency power ratio; ∆ index, index during challenge – index at rest.

**Supplementary Table 4**. Correlations between HRV indices and cognitive change in subjects with NC at baseline (n = 37)

|  | Simple correlation ^a^ | | | Partial correlation ^b^ | | |
| --- | --- | --- | --- | --- | --- | --- |
| Active standing | r | P-value | Q-value ^c^ | r | P-value | Q-value ^c^ |
| ∆ LFn (n.u) ^†^ | 0.128 | 0.450 | 0.655 | 0.092 | 0.630 | 0.720 |
| ∆ LF/HF ^†^ | -0.298 | 0.073 | 0.130 | -0.072 | 0.707 | 0.754 |
| Paced breathing |  |  |  |  |  |  |
| ∆ LFn (n.u) ^‡^ | -0.023 | 0.893 | 0.893 | 0.098 | 0.605 | 0.720 |
| ∆ LF/HF ^‡^ | -0.145 | 0.391 | 0.625 | 0.111 | 0.559 | 0.720 |

**Legend**

^a^ Spearman’s simple correlation between the ∆ HRV index and the annual change in the cognitive Z-score, ^b^ Spearman’s partial correlation adjusted for age, sex, education, physical activity, morbidity (additive index), resting HRV and baseline cognitive Z-score, ^c^ P-value corrected for multiple testing by means of the Benjamini-Hochberg procedure with a 5% False Discovery Rate (FDR) , ^†^ Annual change in cognitive Z-score refers to the episodic memory Z-score (model 1), ^‡^ Annual change in cognitive Z-score refers to the executive functioning Z-score (model 2). Abbreviations: NC, Normal Cognition; r, correlation coefficient; LFn, normalized low frequency power; n.u, normalized units; LF/HF, low frequency power to high frequency power ratio; ∆ index, index during challenge – index at rest.

**Supplementary Table 5**. Correlations between HRV indices and cognitive change in subjects with MCI at baseline (n = 34)

|  | Simple correlation ^a^ | | | Partial correlation ^b^ | | |
| --- | --- | --- | --- | --- | --- | --- |
| Active standing | r | P-value | Q-value ^c^ | r | P-value | Q-value ^c^ |
| ∆ LFn (n.u) ^†^ | -0.499 | **0.003** | **0.006** | -0.436 | **0.023** | **0.046** |
| ∆ LF/HF ^†^ | -0.637 | **< 0.001** | **< 0.001** | -0.650 | **< 0.001** | **< 0.001** |
| Paced breathing |  |  |  |  |  |  |
| ∆ LFn (n.u) ^‡^ | -0.664 | **< 0.001** | **< 0.001** | -0.673 | **< 0.001** | **< 0.001** |
| ∆ LF/HF ^‡^ | -0.608 | **< 0.001** | **< 0.001** | -0.623 | **0.001** | **0.001** |

**Legend**

^a^ Spearman’s simple correlation between the ∆ HRV index and the annual change in the cognitive Z-score, ^b^ Spearman’s partial correlation adjusted for age, sex, education, physical activity, morbidity (additive index), resting HRV and baseline cognitive Z-score, ^c^ P-value corrected for multiple testing by means of the Benjamini-Hochberg procedure with a 5% False Discovery Rate (FDR) , ^†^ Annual change in cognitive Z-score refers to the episodic memory Z-score (model 1), ^‡^ Annual change in cognitive Z-score refers to the executive functioning Z-score (model 2). Significant results are shown in bold typeface. Abbreviations: MCI, Mild Cognitive Impairment; r, correlation coefficient; LFn, normalized low frequency power; n.u, normalized units; LF/HF, low frequency power to high frequency power ratio; ∆ index, index during challenge – index at rest.

**Supplementary Table 6**. Other ∆ HRV indices as predictors of cognitive change in subjects with NC at baseline (n = 37)

|  | Unadjusted model ^a^ | | | Adjusted model ^b^ | | |
| --- | --- | --- | --- | --- | --- | --- |
| Active standing | β | P-value | Q-value ^c^ | β | P-value | Q-value ^c^ |
| ∆ TP (ms^2^) ^†^ | 0.328 | **0.047** | 0.314 | 0.300 | 0.089 | 0.405 |
| ∆ LF (ms^2^) ^†^ | -0.149 | 0.377 | 0.604 | -0.367 | 0.268 | 0.537 |
| ∆ HF (ms^2^) ^†¶^ | -0.104 | 0.547 | 0.673 | -0.004 | 0.995 | 0.995 |
| ∆ SDNN (ms) ^†^**^$^** | -0.279 | 0.111 | 0.405 | 0.149 | 0.406 | 0.628 |
| ∆ RMSSD (ms) ^†^ | -0.112 | 0.511 | 0.673 | -0.093 | 0.718 | 0.789 |
| ∆ pNN50 (%) ^†^ | -0.201 | 0.233 | 0.533 | -0.222 | 0.361 | 0.604 |
| Paced breathing |  |  |  |  |  |  |
| ∆ TP (ms^2^) ^‡^**^§^** | -0.259 | 0.126 | 0.405 | 0.092 | 0.746 | 0.796 |
| ∆ LF (ms^2^) ^‡^**^§^** | 0.278 | 0.101 | 0.405 | -0.179 | 0.591 | 0.709 |
| ∆ HF (ms^2^) ^‡^**^§^** | 0.330 | **0.049** | 0.314 | 0.305 | 0.105 | 0.405 |
| ∆ SDNN (ms) ^‡^ | -0.205 | 0.224 | 0.533 | -0.264 | 0.203 | 0.533 |
| ∆ RMSSD (ms) ^‡^**^§^** | 0.155 | 0.368 | 0.604 | 0.152 | 0.439 | 0.638 |
| ∆ pNN50 (%)^‡^**^§^** | 0.107 | 0.536 | 0.673 | 0.069 | 0.702 | 0.789 |

**Legend**

^a^ Simple linear regression with the ∆ HRV index as independent variable and the annual change in the cognitive Z-score as dependent variable, ^b^ Multiple linear regression adjusted for age, sex, education, physical activity, morbidity (additive index), resting HRV and baseline cognitive Z-score, ^c^ P-value corrected for multiple testing by means of the Benjamini-Hochberg procedure with a 5% False Discovery Rate (FDR), ^†^ Dependent variable: annual change in episodic memory Z-score (model 1), ^‡^ Dependent variable: annual change in executive functioning Z-score (model 2), ^¶^ One outlier removed from the analysis in the unadjusted model, ^$^ Three outliers removed from the analysis in the unadjusted model, **^§^ One outlier removed from the analyses.** Significant results are shown in bold typeface. Abbreviations: NC, Normal Cognition; β, standardized regression coefficient; TP, total power; LF, low frequency power; HF, high frequency power; SDNN, standard deviation of the NN intervals; RMSSD, root mean square of successive differences in the NN intervals; pNN50, percentage of successive NN intervals differing by more than 50 ms; ∆ index, index during challenge – index at rest.

**Supplementary Table 7**. Other ∆ HRV indices as predictors of cognitive change in subjects with MCI at baseline (n = 34)

|  | Unadjusted model ^a^ | | | Adjusted model ^b^ | | |
| --- | --- | --- | --- | --- | --- | --- |
| Active standing | β | P-value | Q-value ^c^ | β | P-value | Q-value ^c^ |
| ∆ TP (ms^2^) ^†¶^ | 0.012 | 0.949 | 0.969 | 0.559 | 0.059 | 0.314 |
| ∆ LF (ms^2^) ^†¶^ | -0.146 | 0.418 | 0.628 | 0.360 | 0.293 | 0.551 |
| ∆ HF (ms^2^) ^†¶^ | 0.182 | 0.310 | 0.551 | 0.068 | 0.699 | 0.789 |
| ∆ SDNN (ms) ^†¶^ | -0.109 | 0.547 | 0.673 | 0.284 | 0.144 | 0.429 |
| ∆ RMSSD (ms) ^†¶^ | -0.120 | 0.507 | 0.673 | 0.165 | 0.475 | 0.671 |
| ∆ pNN50 (%) ^†¶^ | 0.064 | 0.723 | 0.789 | 0.044 | 0.840 | 0.876 |
| Paced breathing |  |  |  |  |  |  |
| ∆ TP (ms^2^) ^‡^ | 0.199 | 0.258 | 0.537 | 0.218 | 0.232 | 0.533 |
| ∆ LF (ms^2^) ^‡^ | -0.251 | 0.152 | 0.429 | -0.583 | **0.006** | 0.136 |
| ∆ HF (ms^2^) ^‡^ | 0.182 | 0.302 | 0.551 | 0.198 | 0.265 | 0.537 |
| ∆ SDNN (ms) ^‡^ | -0.408 | **0.017** | 0.267 | -0.262 | 0.126 | 0.405 |
| ∆ RMSSD (ms) ^‡^ | 0.341 | **0.048** | 0.314 | 0.568 | **0.005** | 0.136 |
| ∆ pNN50 (%) ^‡^ | 0.335 | 0.053 | 0.314 | 0.436 | **0.050** | 0.314 |

**Legend**

^a^ Simple linear regression with the ∆ HRV index as independent variable and the annual change in the cognitive Z-score as dependent variable, ^b^ Multiple linear regression adjusted for age, sex, education, physical activity, morbidity (additive index), resting HRV and baseline cognitive Z-score, ^c^ P-value corrected for multiple testing by means of the Benjamini-Hochberg procedure with a 5% False Discovery Rate (FDR), ^†^ Dependent variable: annual change in episodic memory Z-score (model 1), ^‡^ Dependent variable: annual change in executive functioning Z-score (model 2), ^¶^ One outlier removed from the analyses. Significant results are shown in bold typeface. Abbreviations: MCI, Mild Cognitive Impairment; β, standardized regression coefficient; TP, total power; LF, low frequency power; HF, high frequency power; SDNN, standard deviation of the NN intervals; RMSSD, root mean square of successive differences in the NN intervals; pNN50, percentage of successive NN intervals differing by more than 50 ms; ∆ index, index during challenge – index at rest.

**Supplementary Table 8**. Resting HRV indices as predictors of cognitive change in subjects with NC at baseline (n = 37)

|  | Unadjusted model ^a^ | | | Adjusted model ^b^ | | |
| --- | --- | --- | --- | --- | --- | --- |
|  | β | P-value | Q-value ^c^ | β | P-value | Q-value ^c^ |
| LFn (n.u) | -0.219 | 0.193 | 0.616 | -0.137 | 0.361 | 0.700 |
| LF/HF | -0.181 | 0.283 | 0.670 | -0.073 | 0.648 | 0.814 |
| TP (ms^2^) ^†^ | 0.133 | 0.455 | 0.747 | 0.081 | 0.583 | 0.812 |
| LF (ms^2^) ^†^ | 0.028 | 0.877 | 0.952 | 0.118 | 0.432 | 0.736 |
| HF (ms^2^) ^†^ | 0.003 | 0.985 | 0.985 | 0.036 | 0.815 | 0.917 |
| SDNN (ms) ^†^ | 0.094 | 0.597 | 0.813 | 0.011 | 0.941 | 0.956 |
| RMSSD (ms) ^†^ | -0.052 | 0.768 | 0.894 | 0.012 | 0.937 | 0.956 |
| pNN50 (%)^†^ | -0.016 | 0.928 | 0.956 | 0.063 | 0.668 | 0.823 |
|  | Unadjusted model ^d^ | | | Adjusted model ^e^ | | |
|  | β | P-value | Q-value ^c^ | β | P-value | Q-value ^c^ |
| LFn (n.u) ^‡^ | 0.166 | 0.333 | 0.683 | 0.210 | 0.223 | 0.620 |
| LF/HF ^‡^ | 0.096 | 0.577 | 0.812 | 0.269 | 0.137 | 0.516 |
| TP (ms^2^) ^‡^ | 0.343 | **0.041** | 0.432 | 0.264 | 0.106 | 0.498 |
| LF (ms^2^) ^‡^ | 0.360 | **0.031** | 0.432 | 0.297 | 0.077 | 0.493 |
| HF (ms^2^) ^‡^ | 0.223 | 0.191 | 0.616 | 0.146 | 0.391 | 0.736 |
| SDNN (ms) ^‡^ | 0.190 | 0.267 | 0.657 | 0.107 | 0.518 | 0.790 |
| RMSSD (ms) ^‡^ | 0.134 | 0.437 | 0.736 | 0.079 | 0.638 | 0.814 |
| pNN50 (%) ^‡^ | 0.141 | 0.411 | 0.736 | 0.082 | 0.629 | 0.814 |

**Legend**

^a^ Simple linear regression with the resting HRV index as independent variable and the annual change in the episodic memory Z-score as dependent variable, ^b^ Multiple linear regression adjusted for age, sex, education, physical activity, morbidity (additive index) and baseline episodic memory Z-score, ^c^ P-value corrected for multiple testing by means of the Benjamini-Hochberg procedure with a 5% False Discovery Rate (FDR), ^d^ Simple linear regression with the resting HRV index as independent variable and the annual change in the executive functioning Z-score as dependent variable, ^e^ Multiple linear regression adjusted for age, sex, education, physical activity, morbidity (additive index) and baseline executive functioning Z-score, ^†^ Three outliers removed from the analysis in the unadjusted model, ^‡^ **One outlier removed from the analyses.** Significant results are shown in bold typeface. Abbreviations: NC, Normal Cognition; β, standardized regression coefficient; LFn, normalized low frequency power; n.u, normalized units; LF/HF, low frequency power to high frequency power ratio; TP, total power; LF, low frequency power; HF, high frequency power; SDNN, standard deviation of the NN intervals; RMSSD, root mean square of successive differences in the NN intervals; pNN50, percentage of successive NN intervals differing by more than 50 ms.

**Supplementary Table 9**. Resting HRV indices as predictors of cognitive change in subjects with MCI at baseline (n = 34)

|  | Unadjusted model ^a^ | | | Adjusted model ^b^ | | |
| --- | --- | --- | --- | --- | --- | --- |
|  | β | P-value | Q-value ^c^ | β | P-value | Q-value ^c^ |
| LFn (n.u) ^†^ | 0.226 | 0.205 | 0.620 | 0.137 | 0.420 | 0.736 |
| LF/HF ^†^ | 0.383 | **0.028** | 0.432 | 0.179 | 0.332 | 0.683 |
| TP (ms^2^) ^†^ | 0.254 | 0.154 | 0.548 | 0.287 | 0.091 | 0.498 |
| LF (ms^2^) ^†^ | 0.319 | 0.071 | 0.493 | 0.354 | **0.038** | 0.432 |
| HF (ms^2^) ^†^ | 0.205 | 0.253 | 0.649 | 0.275 | 0.125 | 0.498 |
| SDNN (ms) ^†^ | 0.420 | **0.015** | 0.432 | 0.464 | **0.004** | 0.282 |
| RMSSD (ms) ^†^ | 0.283 | 0.110 | 0.498 | 0.213 | 0.222 | 0.620 |
| pNN50 (%) ^†^ | 0.130 | 0.472 | 0.756 | 0.039 | 0.825 | 0.917 |
|  | Unadjusted model ^d^ | | | Adjusted model ^e^ | | |
|  | β | P-value | Q-value ^c^ | β | P-value | Q-value ^c^ |
| LFn (n.u) | 0.277 | 0.112 | 0.498 | 0.265 | 0.117 | 0.498 |
| LF/HF | 0.170 | 0.336 | 0.683 | 0.039 | 0.831 | 0.917 |
| TP (ms^2^) | 0.023 | 0.899 | 0.956 | 0.058 | 0.747 | 0.886 |
| LF (ms^2^) | 0.088 | 0.622 | 0.814 | 0.111 | 0.536 | 0.797 |
| HF (ms^2^) | 0.072 | 0.687 | 0.829 | 0.179 | 0.342 | 0.683 |
| SDNN (ms) | 0.319 | 0.066 | 0.493 | 0.336 | 0.057 | 0.493 |
| RMSSD (ms) | -0.115 | 0.517 | 0.790 | -0.118 | 0.559 | 0.812 |
| pNN50 (%) | -0.174 | 0.326 | 0.683 | -0.215 | 0.246 | 0.649 |

**Legend**

^a^ Simple linear regression with the resting HRV index as independent variable and the annual change in the episodic memory Z-score as dependent variable, ^b^ Multiple linear regression adjusted for age, sex, education, physical activity, morbidity (additive index) and baseline episodic memory Z-score, ^c^ P-value corrected for multiple testing by means of the Benjamini-Hochberg procedure with a 5% False Discovery Rate (FDR), ^d^ Simple linear regression with the resting HRV index as independent variable and the annual change in the executive functioning Z-score as dependent variable, ^e^ Multiple linear regression adjusted for age, sex, education, physical activity, morbidity (additive index) and baseline executive functioning Z-score, ^†^ One outlier removed from the analyses. Significant results are shown in bold typeface. Abbreviations: MCI, Mild Cognitive Impairment; β, standardized regression coefficient; LFn, normalized low frequency power; n.u, normalized units; LF/HF, low frequency power to high frequency power ratio; TP, total power; LF, low frequency power; HF, high frequency power; SDNN, standard deviation of the NN intervals; RMSSD, root mean square of successive differences in the NN intervals; pNN50, percentage of successive NN intervals differing by more than 50 ms.

**Supplementary Table 10**. Main HRV indices in subjects with NC and MCI at baseline taking part in the follow-up

|  | NC (n = 37) ^†^ | MCI (n =34) ^‡^ |
| --- | --- | --- |
| LFn (n.u) |  |  |
| Resting | 49.4 (17.8) | 49.7 (19.8) |
| Standing | 65.7 (17.6) | 51.4 (19.9) |
| Paced breathing | 37.8 (18.6) | 48.7 (19.5) |
| ∆ Standing | 16.3 (16.2) | 1.7 (25.4) |
| ∆ Paced breathing | -11.7 (19.8) | -1.0 (23.5) |
| LF/HF |  |  |
| Resting | 2.5 (2.2) | 2.4 (2.0) |
| Standing | 5.3 (3.9) | 3.1 (2.8) |
| Paced breathing | 1.2 (1.5) | 1.8 (1.6) |
| ∆ Standing | 2.9 (2.7) | 0.6 (2.7) |
| ∆ Paced breathing | -1.3 (2.0) | -0.6 (2.3) |

**Legend**

HRV indices reported as mean (standard deviation). † 3 subjects lost to follow-up, ^‡^ 6 subjects lost to follow-up. Abbreviations: NC, Normal Cognition; MCI, Mild Cognitive Impairment; LFn, normalized low frequency power; n.u, normalized units; LF/HF, low frequency power to high frequency power ratio; ∆ index, index during challenge – index at rest.

**Supplementary Table 11**. Other HRV indices in subjects with NC and MCI at baseline taking part in the follow-up

|  | NC (n = 37) ^†^ | MCI (n =34) ^‡^ |
| --- | --- | --- |
| TP (ms^2^) |  |  |
| Resting | 1693.7 (4121.6) | 1363.3 (1221.7) |
| Standing | 1353.5 (2503.8) | 981.1 (780.4) |
| Paced breathing | 1903.8 (2442.3) | 1921.4 (1955.3) |
| ∆ Standing | -340.2 (3403.8) | -382.2 (1385.2) |
| ∆ Paced breathing | 210.1 (3269.2) | 558.1 (1904.8) |
| LF (ms^2^) |  |  |
| Resting | 420.3 (964.7) | 284.0 (373.5) |
| Standing | 261.3 (592.2) | 175.2 (202.9) |
| Paced breathing | 611.9 (2546.2) | 437.0 (680.3) |
| ∆ Standing | -159.0 (480.1) | -108.8 (406.3) |
| ∆ Paced breathing | 191.6 (1697.9) | 153.1 (384.8) |
| HF (ms^2^) |  |  |
| Resting | 227.5 (453.9) | 147.3 (130.3) |
| Standing | 75.8 (134.2) | 135.8 (200.7) |
| Paced breathing | 324.7 (615.8) | 319.9 (339.4) |
| ∆ Standing | -151.7 (406.5) | -11.5 (181.1) |
| ∆ Paced breathing | 97.2 (322.7) | 172.6 (296.0) |
| SDNN (ms) |  |  |
| Resting | 38.6 (24.1) | 33.2 (14.9) |
| Standing | 33.2 (21.7) | 28.3 (12.9) |
| Paced breathing | 49.5 (29.9) | 39.2 (23.4) |
| ∆ Standing | -5.4 (11.4) | -4.9 (14.3) |
| ∆ Paced breathing | 10.9 (32.7) | 5.9 (20.5) |
| RMSSD (ms) |  |  |
| Resting | 27.7 (26.0) | 24.3 (17.9) |
| Standing | 19.4 (16.3) | 22.1 (13.2) |
| Paced breathing | 31.6 (26.2) | 27.3 (17.3) |
| ∆ Standing | -8.4 (19.9) | -2.3 (12.4) |
| ∆ Paced breathing | 3.9 (17.6) | 2.9 (18.3) |
| pNN50 (%) |  |  |
| Resting | 4.6 (8.9) | 5.9 (10.0) |
| Standing | 2.7 (6.0) | 4.7 (9.3) |
| Paced breathing | 8.3 (12.6) | 6.4 (9.7) |
| ∆ Standing | -1.9 (8.2) | -1.2 (9.0) |
| ∆ Paced breathing | 3.7 (10.3) | 0.5 (12.6) |

**Legend**

HRV indices reported as mean (standard deviation). † 3 subjects lost to follow-up, ^‡^ 6 subjects lost to follow-up. Abbreviations: NC, Normal Cognition; MCI, Mild Cognitive Impairment; TP, total power; LF, low frequency power; HF, high frequency power; SDNN, standard deviation of the NN intervals; RMSSD, root mean square of successive differences in the NN intervals; pNN50, percentage of successive NN intervals differing by more than 50 ms; ∆ index, index during challenge – index at rest.

**Supplementary Table 12**. Baseline characteristics of the study subjects by cognitive group at follow-up

|  | NC  (n = 26) | MCI  (n = 33) | Dementia  (n = 12) | P-value |
| --- | --- | --- | --- | --- |
| Age (years) | 76.9 (5.0) | 78.2 (5.2) | 80.8 (2.8) | **0.039** ^a^ |
| Sex, female | 22 (84.6) | 24 (72.7) | 9 (75.0) | 0.600 |
| Education (years) | 11.8 (4.6) | 10.7 (4.8) | 10.2 (3.8) | 0.539 |
| BMI (kg/m^2^) | 24.5 (2.5) | 24.7 (3.0) | 23.6 (4.2) | 0.330 |
| Hypertension | 12 (46.2) | 20 (60.6) | 8 (66.7) | 0.394 |
| Smoking | 2 (7.7) | 0 (0.0) | 1 (8.3) | 0.186 |
| Alcohol (AU/day) | 1.4 (1.6) | 1.4 (1.7) | 0.8 (1.0) | 0.594 |
| Coffee (cups/day) | 1.5 (1.1) | 1.6 (1.1) | 1.0 (0.7) | 0.339 |
| Physical activity (MET-hrs/week) | 72.1 (35.0) | 72.2 (44.5) | 32.5 (23.9) | **0.004** ^a,b^ |
| Glucose (mg/dl) | 87.7 (10.1) | 90.5 (9.0) | 87.3 (12.7) | 0.397 |
| Total cholesterol (mg/dl) | 229.8 (33.0) | 221.0 (29.6) | 231.6 (45.0) | 0.708 |
| LDL cholesterol (mg/dl) | 140.8 (33.3) | 141.9 (21.7) | 133.6 (41.6) | 0.293 |
| HDL cholesterol (mg/dl) | 71.8 (20.0) | 64.1 (13.7) | 75.9 (25.6) | 0.218 |
| Triglycerides (mg/dl) | 105.7 (26.5) | 104.7 (34.2) | 101.3 (39.4) | 0.854 |
| Number of medications | 3.1 (1.7) | 3.6 (2.3) | 4.3 (1.6) | 0.181 |
| Antihypertensive medications |  |  |  |  |
| ACE-Is/ARBs | 10 (38.5) | 18 (54.5) | 7 (58.3) | 0.372 |
| Diuretics | 6 (23.1) | 5 (15.2) | 5 (41.7) | 0.179 |
| CCBs (peripheral) | 1 (3.8) | 6 (18.2) | 2 (16.7) | 0.217 |
| Psychotropic medications |  |  |  |  |
| SSRIs | 7 (26.9) | 7 (21.2) | 5 (41.7) | 0.396 |
| Benzodiazepines | 6 (23.1) | 7 (21.2) | 1 (8.3) | 0.621 |
| Scale scores |  |  |  |  |
| BADL score | 5.5 (0.5) | 5.5 (0.5) | 5.5 (0.5) | 0.972 |
| IADL score | 7.5 (1.1) | 7.0 (1.4) | 6.8 (1.5) | 0.193 |
| MMSE score | 28.5 (1.1) | 27.7 (1.7) | 26.5 (2.1) | **0.015** ^a^ |
| CIRS-m score | 2.2 (1.3) | 2.0 (1.3) | 1.8 (1.1) | 0.575 |
| STPI-TA score | 19.8 (6.3) | 20.6 (5.3) | 17.6 (6.3) | 0.111 |
| GDS-s score | 2.9 (2.4) | 3.9 (3.6) | 2.7 (2.1) | 0.722 |

**Legend**

Continuous variables reported as mean (standard deviation) and compared with the Kruskall-Wallis test with Bonferroni-corrected pairwise comparisons. Categorical variables reported as n (%) and compared with the Chi-squared test or Fisher’s exact test. Significant results are shown in bold typeface. ^a^ Significant difference between NC and dementia, ^b^ Significant difference between MCI and dementia. Abbreviations: NC, Normal Cognition; MCI, Mild Cognitive Impairment; BMI, Body Mass Index; AU, Alcohol Units; MET, Metabolic Equivalent; LDL, Low Density Lipoproteins; HDL, High Density Lipoproteins; ACE-Is, Angiotensin Converting Enzyme Inhibitors; ARBs, Angiotensin Receptor Blockers; SSRIs, Selective Serotonin Reuptake Inhibitors; CCBs, Calcium Channel Blockers; BADL, Basic Activities of Daily Living; IADL, Instrumental Activities of Daily Living; MMSE, Mini Mental State Examination; CIRS-m, Cumulative Illness Rating score- morbidity; STPI-TA, State Trait Personality Inventory- Trait anxiety; GDS-s, short Geriatric Depression Scale.

**Supplementary Table 13**. Cognitive and functional status of the study subjects at follow-up

| Neuropsychological test | NC  (n = 26) | MCI  (n = 33) | Dementia  (n = 12) | P-value |
| --- | --- | --- | --- | --- |
| Memory |  |  |  |  |
| Prose recall | 12.8 (2.2) | 10.1 (3.4) | 3.5 (4.7) | **< 0.001** ^a,b,c^ |
| ROCF-delayed recall | 22.0 (6.6) | 15.7 (8.1) | 6.1 (9.3) | **< 0.001** ^a,b,c^ |
| Executive functions |  |  |  |  |
| Digit Cancellation Test | 54.9 (5.4) | 49.0 (6.5) | 41.9 (9.9) | **< 0.001** ^a,c^ |
| Bell test | 34.2 (0.8) | 32.9 (2.1) | 30.9 (4.8) | **0.002** ^a,c^ |
| Digit Span Forwards | 6.0 (0.9) | 5.0 (1.2) | 4.5 (1.6) | **< 0.001** ^a,c^ |
| Digit Span Backwards | 4.6 (0.7) | 4.1 (1.1) | 2.9 (1.6) | **0.001** ^a,b^ |
| Trail-Making Test A | 26.8 (12.9) | 35.6 (15.4) | 89.5 (59.2) | **< 0.001** ^a,b^ |
| Trail-Making Test B | 51.1 (35.3) | 161.7 (136.2) | 367.8 (116.2) | **< 0.001** ^a,b,c^ |
| Weigl's Test | 11.9 (2.1) | 9.7 (2.7) | 6.8 (2.8) | **< 0.001** ^a,c^ |
| Cognitive Estimates-total | 12.7 (2.5) | 17.2 (2.6) | 19.4 (3.8) | **< 0.001** ^a,c^ |
| Cognitive Estimates-bizarre | 2.6 (1.6) | 4.8 (1.6) | 6.3 (2.6) | **< 0.001** ^a,c^ |
| Raven's CPM | 33.0 (4.0) | 28.3 (6.0) | 31.6 (26.7) | **< 0.001** ^a,c^ |
| Letter fluency | 35.8 (6.9) | 31.8 (9.2) | 18.3 (7.1) | **< 0.001** ^a,b^ |
| Language |  |  |  |  |
| Category fluency | 19.9 (3.6) | 15.3 (3.8) | 10.0 (2.9) | **< 0.001** ^a,b,c^ |
| Picture naming | 76.0 (2.9) | 70.2 (5.1) | 62.4 (9.4) | **< 0.001** ^a,c^ |
| Token Test | 34.0 (2.2) | 31.2 (2.5) | 27.9 (2.0) | **< 0.001** ^a,b,c^ |
| Visuospatial skills |  |  |  |  |
| ROCF-copy | 35.5 (1.8) | 31.9 (4.2) | 25.8 (7.5) | **< 0.001** ^a,c^ |
| Copy of geometric figures | 13.8 (0.4) | 12.8 (1.2) | 11.0 (3.9) | **< 0.001** ^a,c^ |
| Ideomotor praxis |  |  |  |  |
| De Renzi's Test-right upper limb | 71.7 (0.6) | 70.5 (1.9) | 67.8 (5.2) | **< 0.001** ^a,c^ |
| De Renzi's Test-left upper limb | 71.6 (0.9) | 70.5 (2.5) | 68.2 (4.5) | **0.001** ^a^ |
| Scale scores |  |  |  |  |
| BADL score | 5.5 (0.5) | 5.4 (0.5) | 5.2 (0.8) | 0.575 |
| IADL score | 7.4 (1.2) | 6.6 (1.4) | 4.2 (2.3) | **< 0.001** ^a,b^ |
| MMSE score | 28.5 (1.4) | 27.8 (2.1) | 21.8 (4.2) | **< 0.001** ^a,b^ |

**Legend**

Variables reported as mean (standard deviation) and compared with the Kruskall-Wallis test with Bonferroni-corrected pairwise comparisons. Neuropsychological test scores and the MMSE score are demographically-adjusted. Higher scores indicate better performance except for the Trail-Making and Cognitive Estimates tests for which the reverse applies. Significant results are shown in bold typeface. ^a^ Significant difference between NC and dementia, ^b^ Significant difference between MCI and dementia, ^c^ Significant difference between NC and MCI. Abbreviations: NC, Normal Cognition; MCI, Mild Cognitive Impairment; ROCF, Rey-Osterrieth Complex Figure; CPM, Colored Progressive Matrices; BADL, Basic Activities of Daily Living; IADL, Instrumental Activities of Daily Living; MMSE, Mini Mental State Examination.

**Supplementary Table 14**. HRV indices according to mortality status at follow-up

|  | No mortality  (n = 73) | Mortality  (n = 7) | P-value ^a^ | Q-value ^b^ |
| --- | --- | --- | --- | --- |
| Active standing |  |  |  |  |
| ∆ LFn (n.u) | 9.2 (22.2) | 22.8 (24.9) | 0.243 | 0.373 |
| ∆ LF/HF | 1.7 (3.0) | 2.1 (2.9) | 0.865 | 0.865 |
| Paced breathing |  |  |  |  |
| ∆ LFn (n.u) | -7.2 (21.8) | 12.6 (20.8) | **0.026** | 0.103 |
| ∆ LF/HF | -1.1 (2.5) | -0.5 (2.3) | 0.280 | 0.373 |

**Legend**

HRV indices reported as mean (standard deviation). ^a^ Mann-Whitney’s U test, ^b^ P-value corrected for multiple testing by means of the Benjamini-Hochberg procedure with a 5% False Discovery Rate (FDR). Significant results are shown in bold typeface. Abbreviations: LFn, normalized low frequency power; n.u, normalized units; LF/HF, low frequency power to high frequency power ratio; ∆ index, index during challenge – index at rest.

**Supplementary Table 15**. Associations between HRV indices and cognitive performance at baseline in subjects with NC (n = 40)

|  | Unadjusted model ^a^ | | | Adjusted model ^b^ | | |
| --- | --- | --- | --- | --- | --- | --- |
| Active standing | β | P-value | Q-value ^c^ | β | P-value | Q-value ^c^ |
| ∆ LFn (n.u) ^†^ | 0.077 | 0.635 | 0.923 | 0.244 | 0.206 | 0.799 |
| ∆ LF/HF ^†^ | 0.320 | **0.044** | 0.702 | 0.258 | 0.140 | 0.799 |
| Paced breathing |  |  |  |  |  |  |
| ∆ LFn (n.u) ^‡^ | -0.128 | 0.431 | 0.799 | 0.044 | 0.827 | 0.955 |
| ∆ LF/HF ^‡^ | -0.096 | 0.557 | 0.891 | 0.226 | 0.330 | 0.799 |

**Legend**

^a^ Simple linear regression with the ∆ HRV index as independent variable and the cognitive Z-score at baseline as dependent variable, ^b^ Multiple linear regression adjusted for age, sex, education, physical activity, morbidity (additive index) and resting HRV, ^c^ P-value corrected for multiple testing by means of the Benjamini-Hochberg procedure with a 5% False Discovery Rate (FDR), ^†^ Dependent variable: episodic memory Z-score at baseline (model 1), ^‡^ Dependent variable: executive functioning Z-score at baseline (model 2). Significant results are shown in bold typeface. Abbreviations: NC, Normal Cognition; β, standardized regression coefficient; LFn, normalized low frequency power; n.u, normalized units; LF/HF, low frequency power to high frequency power ratio; ∆ index, index during challenge – index at rest.

**Supplementary Table 16**. Associations between HRV indices and cognitive performance at baseline in subjects with MCI (n = 40)

|  | Unadjusted model ^a^ | | | Adjusted model ^b^ | | |
| --- | --- | --- | --- | --- | --- | --- |
| Active standing | β | P-value | Q-value ^c^ | β | P-value | Q-value ^c^ |
| ∆ LFn (n,u) ^†^ | -0.123 | 0.449 | 0.799 | -0.199 | 0.421 | 0.799 |
| ∆ LF/HF ^†^ | -0.009 | 0.955 | 0.955 | -0.020 | 0.915 | 0.955 |
| Paced breathing |  |  |  |  |  |  |
| ∆ LFn (n.u) ^‡^ | -0.012 | 0.943 | 0.955 | -0.013 | 0.951 | 0.955 |
| ∆ LF/HF ^‡^ | -0.149 | 0.358 | 0.799 | -0.260 | 0.409 | 0.799 |

**Legend**

^a^ Simple linear regression with the ∆ HRV index as independent variable and the cognitive Z-score at baseline as dependent variable, ^b^ Multiple linear regression adjusted for age, sex, education, physical activity, morbidity (additive index) and resting HRV , ^c^ P-value corrected for multiple testing by means of the Benjamini-Hochberg procedure with a 5% False Discovery Rate (FDR), ^†^ Dependent variable: episodic memory Z-score at baseline (model 1), ^‡^ Dependent variable: executive functioning Z-score at baseline (model 2). Abbreviations: MCI, Mild Cognitive Impairment; β, standardized regression coefficient; LFn, normalized low frequency power; n.u, normalized units; LF/HF, low frequency power to high frequency power ratio; ∆ index, index during challenge – index at rest.

**Supplementary Table 17**. Cerebrovascular burden and hippocampal atrophy on computed tomography in the NC and MCI subjects at baseline.

|  | NC (n = 40) ^†^ | MCI (n = 40) | P-value |
| --- | --- | --- | --- |
| Cerebrovascular burden ^a^ |  |  |  |
| PV white matter lesions | 1.4 (0.5) | 1.5 (0.6) | 0.720 |
| Deep white matter lesions | 1.3 (0.6) | 1.5 (0.6) | 0.163 |
| Hippocampal atrophy ^b^ | 1.4 (0.5) | 2.0 (0.7) | **0.004** |

**Legend**

Variables reported as mean (standard deviation) and compared with Mann-Whitney’s U-test. Significant results are shown in bold typeface. ^a^ Fazekas’ scale score: higher scores indicate greater cerebrovascular burden, ^b^ Kim’s scale score: higher scores indicate greater hippocampal atrophy, ^†^ Available for n = 16 subjects. Abbreviations: NC, Normal Cognition; MCI, Mild Cognitive Impairment; PV, periventricular.

**Supplementary References**

Aitake, M., Hori, E., Matsumoto, J., Umeno, K., Fukuda, M., Ono, T., et al. (2011). Sensory mismatch induces autonomic responses associated with hippocampal theta waves in rats. *Behav Brain Res*. 220, 244-253. doi: 10.1016/j.bbr.2011.02.011.

Allen, B., Jennings, J. R., Gianaros, P. J., Thayer, J. F., and Manuck, S. B. (2015). Resting high-frequency heart rate variability is related to resting brain perfusion. *Psychophysiology*. 52, 277-287. doi: 10.1111/psyp.12321.

Alvarez, J. A., and Emory, E. (2006). Executive function and the frontal lobes: a meta-analytic review. *Neuropsychol Rev*. 16, 17-42. doi: 10.1007/s11065-006-9002-x.

Alzheimer’s Society. (2020). Alzheimer’s Society’s view on demography. https://[www.alzheimers.org.uk/about-us/policy-and-influencing/what-we-think/demography](http://www.alzheimers.org.uk/about-us/policy-and-influencing/what-we-think/demography) [Accessed February 14, 2022].

Barbarotto, R., Laiacona, M., Frosio, R., Vecchio, M., Farinato, A., and Capitani, E. (1998). A normative study on visual reaction times and two Stroop colour-word tests. *Ital J Neurol Sci*. 19, 161-170. doi: 10.1007/BF00831566.

Bastin, C., and Salmon, E. (2014). Early neuropsychological detection of Alzheimer's disease. *Eur J Clin Nutr*. 68, 1192-9. doi: 10.1038/ejcn.2014.176.

Caffarra, P., Vezzadini, G., Dieci, F., Zonato, F., and Venneri, A. (2002). Rey-Osterrieth complex figure: normative values in an Italian population sample. *Neurol Sci*. 22, 443-447. doi: 10.1007/s100720200003.

Carlesimo, G., Buccione, I., Fadda, L., Graceffa, A., Mauri, M., Lorusso, S., et al. (2002). Normative data of two memory tasks: Short-Story recall and Rey's Figure. *Nuova Rivista di Neurologia*. 12, 1-13.

Chen, J., Shu, H., Wang, Z., Liu, D., Shi, Y., Zhang, X., et al. (2015). The interaction of APOE genotype by age in amnestic mild cognitive impairment: a voxel-based morphometric study. *J Alzheimers Dis*. 43, 657-668. doi: 10.3233/JAD-141677.

Chen, Y., Denny, K. G., Harvey, D., Farias, S. T., Mungas, D., DeCarli, C., et al (2017). Progression from normal cognition to mild cognitive impairment in a diverse clinic-based and community-based elderly cohort. *Alzheimers Dement*. 13, 399-405. doi: 10.1016/j.jalz.2016.07.151.

Chen, H., Su, F., Ye, Q., Wang, Z., Shu, H., and Bai, F. (2018). The Dose-Dependent Effects of Vascular Risk Factors on Dynamic Compensatory Neural Processes in Mild Cognitive Impairment. *Front Aging Neurosci*.10, 131. doi: 10.3389/fnagi.2018.00131.

Chudasama, Y. (2011). Animal models of prefrontal-executive function. *Behav Neurosci*. 125, 327-343. doi: 10.1037/a0023766.

Dahan, L., Rampon, C., and Florian, C. (2020). Age-related memory decline, dysfunction of the hippocampus and therapeutic opportunities. *Prog Neuropsychopharmacol Biol Psychiatry*. 102, 109943. doi: 10.1016/j.pnpbp.2020.109943.

Dahl, M. J., Mather, M., Düzel, S., Bodammer, N. C., Lindenberger, U., Kühn, S., et al. (2019). Rostral locus coeruleus integrity is associated with better memory performance in older adults. *Nat Hum Behav*. 3, 1203-1214. doi: 10.1038/s41562-019-0715-2.

De Renzi, E., Motti, F., and Nichelli, P. (1980). Imitating gestures. A quantitative approach to ideomotor apraxia. *Arch Neurol*. 37, 6-10. doi: 10.1001/archneur.1980.00500500036003.

Della Sala, S., MacPherson, S. E., Phillips, L. H., Sacco, L., and Spinnler, H. (2003). How many camels are there in Italy? Cognitive estimates standardised on the Italian population. *Neurol Sci*. 24, 10-15. doi: 10.1007/s100720300015.

Dickerson, B. C., and Eichenbaum, H. (2010). The episodic memory system: neurocircuitry and disorders. *Neuropsychopharmacology*. 35, 86-104. doi: 10.1038/npp.2009.126.

Dubois, B., Feldman, H. H., Jacova, C., Hampel, H., Molinuevo, J. L., Blennow, K., et al. (2014). Advancing research diagnostic criteria for Alzheimer's disease: the IWG-2 criteria. *Lancet Neurol*. 13, 614-629. doi: 10.1016/S1474-4422(14)70090-0.

Farias, S. T., Mungas, D., Reed, B. R., Harvey, D., and DeCarli, C. (2009). Progression of mild cognitive impairment to dementia in clinic- vs community-based cohorts. *Arch Neurol*. 66, 1151-1157. doi: 10.1001/archneurol.2009.106.

Gallassi, R., Oppi, F., Poda, R., Scortichini, S., Stanzani Maserati, M., Marano, G., et al. (2010). Are subjective cognitive complaints a risk factor for dementia? *Neurol Sci*. 31, 327-336. doi: 10.1007/s10072-010-0224-6.

Gianaros, P. J., Derbyshire, S. W., May, J. C., Siegle, G. J., Gamalo, M. A., and Jennings, J. R. (2005). Anterior cingulate activity correlates with blood pressure during stress. *Psychophysiology*. 42, 627-635. doi: 10.1111/j.1469-8986.2005.00366.x.

Ginty, A. T., Gianaros, P. J., Derbyshire, S. W., Phillips, A. C., and Carroll, D. (2013). Blunted cardiac stress reactivity relates to neural hypoactivation. *Psychophysiology*. 50, 219-229. doi: 10.1111/psyp.12017.

Giovagnoli, A. R., Del Pesce, M., Mascheroni, S., Simoncelli, M., Laiacona, M., and Capitani, E. (1996). Trail making test: normative values from 287 normal adult controls. *Ital J Neurol Sci*. 17, 305-309. doi: 10.1007/BF01997792.

Gold, B. T., Brown, C. A., Hakun, J. G., Shaw, L. M., Trojanowski, J. Q., and Smith, C. D. (2017). Clinically silent Alzheimer's and vascular pathologies influence brain networks supporting executive function in healthy older adults. *Neurobiol Aging*. 58, 102-111. doi: 10.1016/j.neurobiolaging.2017.06.012.

Hämmerer, D., Callaghan, M. F., Hopkins, A., Kosciessa, J., Betts, M., Cardenas-Blanco, A., et al. (2018). Locus coeruleus integrity in old age is selectively related to memories linked with salient negative events. *Proc Natl Acad Sci U S A*. 115, 2228-2233. doi: 10.1073/pnas.1712268115.

Houx, P. J., Shepherd, J., Blauw, G. J., Murphy, M. B., Ford, I., Bollen, E. L., et al. (2002). Testing cognitive function in elderly populations: the PROSPER study. PROspective Study of Pravastatin in the Elderly at Risk. *J Neurol Neurosurg Psychiatry*. 73, 385-389. doi: 10.1136/jnnp.73.4.385.

Howieson, D. (2019). Current limitations of neuropsychological tests and assessment procedures. *Clin Neuropsychol*. 33, 200-208. doi: 10.1080/13854046.2018.1552762.

Hughes, M. L., Agrigoroaei, S., Jeon, M., Bruzzese, M., and Lachman, M. E. (2018). Change in Cognitive Performance From Midlife Into Old Age: Findings from the Midlife in the United States (MIDUS) Study. *J Int Neuropsychol Soc*. 24, 805-820. doi: 10.1017/S1355617718000425.

Istat. (2021). Demografia in cifre. <https://demo.istat.it> [Accessed February 14, 2022].

Jack C.R. Jr., Bennett, D. A., Blennow, K., Carrillo, M. C., Dunn, B., Haeberlein, S. B., et al. (2018). NIA-AA Research Framework: Toward a biological definition of Alzheimer's disease. *Alzheimers Dement*. 14, 535-562. doi: 10.1016/j.jalz.2018.02.018.

Kesner, R. P., and Churchwell, J. C. (2011). An analysis of rat prefrontal cortex in mediating executive function. *Neurobiol Learn Mem*. 96, 417-431. doi: 10.1016/j.nlm.2011.07.002.

Khookhor, O., and Umegaki, H. (2013). The cholinergic stimulation of the hippocampus induced the activation of the sympathetic nervous system. *Neuro Endocrinol Lett*. 34, 58-61.

Kimmerly, D. S. (2017). A review of human neuroimaging investigations involved with central autonomic regulation of baroreflex-mediated cardiovascular control. *Auton Neurosci*. 207, 10-21. doi: 10.1016/j.autneu.2017.05.008.

Knopman, D. S., DeKosky, S. T., Cummings, J. L., Chui, H., Corey-Bloom, J., Relkin, N., et al. (2010). Practice parameter: diagnosis of dementia (an evidence-based review). Report of the Quality Standards Subcommittee of the American Academy of Neurology. *Neurology*. 56, 1143-1153. doi: 10.1212/wnl.56.9.1143.

Kurth, F., Zilles, K., Fox, P. T., Laird, A. R., and Eickhoff, S. B. (2010). A link between the systems: functional differentiation and integration within the human insula revealed by meta-analysis. *Brain Struct Funct*. 214, 519-534. doi: 10.1007/s00429-010-0255-z.

Laiacona, M., Barbarotto, R., Trivelli, C., and Capitani, E. (1993). Dissociazioni semantiche intercategoriali: descrizione di una batteria standardizzata e dati normativi. *Arch Psicol Neurol.* 54, 209-248.

Lathers, C. M., Schraeder, P. L., and Tumer, N. (1993). The effect of phenobarbital on autonomic function and epileptogenic activity induced by the hippocampal injection of penicillin in cats. *J Clin Pharmacol*. 33, 837-844. doi: 10.1002/j.1552-4604.1993.tb01960.x.

Lonie, J. A., Tierney, K. M., and Ebmeier, K. P. (2009). Screening for mild cognitive impairment: a systematic review. *Int J Geriatr Psychiatry*. 24, 902-915. doi: 10.1002/gps.2208.

Machetanz, K., Berelidze, L., Guggenberger, R., and Gharabaghi, A. (2021). Brain-Heart Interaction During Transcutaneous Auricular Vagus Nerve Stimulation. *Front Neurosci*. 15, 632697. doi: 10.3389/fnins.2021.632697.

Maioli, F., Coveri, M., Pagni, P., Chiandetti, C., Marchetti, C., Ciarrocchi, R., et al. (2007). Conversion of mild cognitive impairment to dementia in elderly subjects: a preliminary study in a memory and cognitive disorder unit. *Arch Gerontol Geriatr*. 44, 233-241. doi: 10.1016/j.archger.2007.01.032.

Marshall, G. A., Amariglio, R. E., Sperling, R. A., and Rentz, D. M. (2012). Activities of daily living: where do they fit in the diagnosis of Alzheimer's disease? *Neurodegener Dis Manag*. 2, 483-491. doi: 10.2217/nmt.12.55.

Mather, M., and Harley, C. W. (2016). The Locus Coeruleus: Essential for Maintaining Cognitive Function and the Aging Brain. *Trends Cogn Sci*. 20, 214-226. doi: 10.1016/j.tics.2016.01.001.

Mather, M., Joo Yoo, H., Clewett, D. V., Lee, T. H., Greening, S. G., Ponzio, A., et al. (2017). Higher locus coeruleus MRI contrast is associated with lower parasympathetic influence over heart rate variability. *Neuroimage*. 150, 329-335. doi: 10.1016/j.neuroimage.2017.02.025.

Mather, M., and Thayer, J. (2018). How heart rate variability affects emotion regulation brain networks. *Curr Opin Behav Sci*. 19, 98-104. doi: 10.1016/j.cobeha.2017.12.017.

McNamara, C. G., and Dupret, D. (2017). Two sources of dopamine for the hippocampus. *Trends Neurosci*. 40, 383-384. doi: 10.1016/j.tins.2017.05.005.

Michaud, T. L., Su, D., Siahpush, M., and Murman, D. L. (2017). The Risk of Incident Mild Cognitive Impairment and Progression to Dementia Considering Mild Cognitive Impairment Subtypes. *Dement Geriatr Cogn Dis Extra*. 7, 15-29. doi: 10.1159/000452486.

Monaco, M., Costa, A., Caltagirone, C., and Carlesimo, G. A. (2013). Forward and backward span for verbal and visuo-spatial data: standardization and normative data from an Italian adult population. *Neurol Sci*. 34, 749-754. doi: 10.1007/s10072-012-1130-x.

Moraes-Neto, T. B., Scopinho, A. A., Biojone, C., Corrêa, F. M., and Resstel, L. B. (2014). Involvement of dorsal hippocampus glutamatergic and nitrergic neurotransmission in autonomic responses evoked by acute restraint stress in rats. *Neuroscience*. 258, 364-373. doi: 10.1016/j.neuroscience.2013.11.022.

Nagai, Y., Critchley, H. D., Featherstone, E., Trimble, M. R., and Dolan, R. J. (2004). Activity in ventromedial prefrontal cortex covaries with sympathetic skin conductance level: a physiological account of a "default mode" of brain function. *Neuroimage*. 22, 243-251. doi: 10.1016/j.neuroimage.2004.01.019.

National Institute for Clinical Excellence (NICE). (2011). Donepezil, Galantamine, Rivastigmine and Memantine for the Treatment of Alzheimer’s Disease. Technology Appraisal Guidance 217. https://publications.nice.org.uk/ donepezil-galantamine-rivastigmine-and-memantine-for-the-treatmentof-alzheimers-disease-ta217/guidance [Accessed February 14, 2022].

Nikolin, S., Boonstra, T. W., Loo, C. K., and Martin, D. (2017). Combined effect of prefrontal transcranial direct current stimulation and a working memory task on heart rate variability. *PLoS One*. 12, e0181833. doi: 10.1371/journal.pone.0181833.

Novelli, G., Papagno, C., Capitani, E., and Laiacona, M. (1986). Tre test clinici di memoria verbale a lungo termine: Taratura su soggetti normali [Three clinical tests for the assessment of verbal long-term memory function: Norms from 320 normal subjects]. *Arch Psicol Neurol Psichiatr*. 47, 278–296.

Nyberg, L. (2017). Functional brain imaging of episodic memory decline in ageing. *J Intern Med*. 281, 65-74. doi: 10.1111/joim.12533.

Orsini, A., Grossi, D., Capitani, E., Laiacona, M., Papagno, C., and Vallar, G. (1987). Verbal and spatial immediate memory span: normative data from 1355 adults and 1112 children. *Ital J Neurol* Sci. 8, 539-548. doi: 10.1007/BF02333660.

Owens, N. C., and Verberne, A. J. (2001). Regional haemodynamic responses to activation of the medial prefrontal cortex depressor region. *Brain Res*. 919, 221-231. doi: 10.1016/s0006-8993(01)03017-7.

Patron, E., Mennella, R., Messerotti Benvenuti, S., and Thayer, J. F. (2019). The frontal cortex is a heart-brake: Reduction in delta oscillations is associated with heart rate deceleration. *Neuroimage*. 188, 403-410. doi: 10.1016/j.neuroimage.2018.12.035.

Rostamian, S., van Buchem, M. A., Westendorp, R. G., Jukema, J. W., Mooijaart, S. P., et al. (2015). Executive function, but not memory, associates with incident coronary heart disease and stroke. *Neurology*. 85, 783-789. doi: 10.1212/WNL.0000000000001895.

Ruggiero, D. A., Mraovitch, S., Granata, A. R., Anwar, M., and Reis, D. J. (1987). A role of insular cortex in cardiovascular function*. J Comp Neurol*. 257, 189-207. doi: [10.1002/cne.902570206](https://doi.org/10.1002/cne.902570206).

Ruit, K. G., and Neafsey, E. J. (1988). Cardiovascular and respiratory responses to electrical and chemical stimulation of the hippocampus in anesthetized and awake rats. *Brain Res*. 457, 310-321. doi: 10.1016/0006-8993(88)90701-9.

Samuels, E. R., and Szabadi, E. (2008). Functional neuroanatomy of the noradrenergic locus coeruleus: its roles in the regulation of arousal and autonomic function part I: principles of functional organisation. *Curr Neuropharmacol*. 6, 235-253. doi: 10.2174/157015908785777229.

Schumann, A., de la Cruz, F., Köhler, S., Brotte, L., and Bär, K. J. (2021). The Influence of Heart Rate Variability Biofeedback on Cardiac Regulation and Functional Brain Connectivity. *Front Neurosci*. 15, 691988. doi: 10.3389/fnins.2021.691988.

Sclocco, R., Beissner, F., Desbordes, G., Polimeni, J. R., Wald, L. L., Kettner, N. W., et al. (2016). Neuroimaging brainstem circuitry supporting cardiovagal response to pain: a combined heart rate variability/ultrahigh-field (7 T) functional magnetic resonance imaging study. *Philos Trans A Math Phys Eng Sci*. 374, 20150189. doi: 10.1098/rsta.2015.0189.

Seo, E. H., and Choo, I. L., Alzheimer’s Disease Neuroimaging Initiative. (2016). Amyloid-independent functional neural correlates of episodic memory in amnestic mild cognitive impairment. *Eur J Nucl Med Mol Imaging*. 43, 1088-1095. doi: 10.1007/s00259-015-3261-9.

Singh-Manoux, A., Kivimaki, M., Glymour, M. M., Elbaz, A., Berr, C., Ebmeier, K. P., et al. (2012). Timing of onset of cognitive decline: results from Whitehall II prospective cohort study. *BMJ*. 344, d7622. doi: 10.1136/bmj.d7622.

Smith, E. E. (2017). Clinical presentations and epidemiology of vascular dementia. *Clin Sci (Lond)*. 131, 1059-1068. doi: 10.1042/CS20160607.

Spaniol, J., Davidson, P. S., Kim, A. S., Han, H., Moscovitch, M., and Grady, C. L. (2009). Event-related fMRI studies of episodic encoding and retrieval: meta-analyses using activation likelihood estimation. *Neuropsychologia*. 47, 1765-1779. doi: 10.1016/j.neuropsychologia.2009.02.028.

Spinnler, H., and Tognoni, G. (1987). Italian standardization and classification of Neuropsychological tests. The Italian Group on the Neuropsychological Study of Aging. *J Neurol Sci.* 8, 1-120.

Staresina, B. P., and Wimber, M. A (2019). Neural Chronometry of Memory Recall. *Trends Cogn Sci*. 23, 1071-1085. doi: 10.1016/j.tics.2019.09.011.

Sun, M. K. (1992). Medullospinal vasomotor neurones mediate hypotension from stimulation of prefrontal cortex. *J Auton Nerv Syst*. 38, 209-217. doi: 10.1016/0165-1838(92)90032-c.

Theofilas, P., Ehrenberg, A. J., Dunlop, S., Di Lorenzo Alho, A. T., Nguy, A., et al. (2017). Locus coeruleus volume and cell population changes during Alzheimer's disease progression: A stereological study in human postmortem brains with potential implication for early-stage biomarker discovery. *Alzheimers Dement*. 13, 236-246. doi: 10.1016/j.jalz.2016.06.2362.

Valenza, G., Allegrini, P., Lanatà, A., and Scilingo, E. P. (2012). Dominant Lyapunov exponent and approximate entropy in heart rate variability during emotional visual elicitation. *Front Neuroeng*. 5, 3. doi: 10.3389/fneng.2012.00003.

Valenza, G., Sclocco, R., Duggento, A., Passamonti, L., Napadow, V., Barbieri, R., et al. (2019). The central autonomic network at rest: Uncovering functional MRI correlates of time-varying autonomic outflow. *Neuroimage*. 197, 383-390. doi: 10.1016/j.neuroimage.2019.04.075.

Valenza, G., Passamonti, L., Duggento, A., Toschi, N., and Barbieri, R. (2020). Uncovering complex central autonomic networks at rest: a functional magnetic resonance imaging study on complex cardiovascular oscillations. *J R Soc Interface*. 17, 20190878. doi: 10.1098/rsif.2019.0878.

Vallar, G., Rusconi, M. L., Fontana, S., and Musicco, M. (1994). Three clinical tests for the assessment of visuospatial exploration. Norms from 212 normal subjects. *Arch Psicol Neurol Psichiatr*. 54, 827-841.

Van der Elst, W., van Boxtel, M. P., van Breukelen, G. J., and Jolles, J. (2005). Rey's verbal learning test: normative data for 1855 healthy participants aged 24-81 years and the influence of age, sex, education, and mode of presentation. *J Int Neuropsychol Soc*. 11, 290-302. doi: 10.1017/S1355617705050344.

Van der Elst, W., van Boxtel, M. P., van Breukelen, G. J., and Jolles, J. (2006). The Letter Digit Substitution Test: normative data for 1,858 healthy participants aged 24-81 from the Maastricht Aging Study (MAAS): influence of age, education, and sex. *J Clin Exp Neuropsychol*. 28, 998-1009. doi: 10.1080/13803390591004428.

Veldsman, M., Tai, X. Y., Nichols, T., Smith, S., Peixoto, J., Manohar, S., et al. (2020). Cerebrovascular risk factors impact frontoparietal network integrity and executive function in healthy ageing. *Nat Commun*. 11, 4340. doi: 10.1038/s41467-020-18201-5.

Wei, L., Chen, H., and Wu, G. R. (2018). Heart rate variability associated with grey matter volumes in striatal and limbic structures of the central autonomic network. *Brain Res*. 1681, 14-20. doi: 10.1016/j.brainres.2017.12.024.

Weissman, D. G., Guyer, A. E., Ferrer, E., Robins, R. W., and Hastings, P. D. (2018). Adolescents' brain-autonomic coupling during emotion processing. *Neuroimage*. 183, 818-827. doi: 10.1016/j.neuroimage.2018.08.069.

Wood, K. N., Badrov, M. B., Speechley, M. R., and Shoemaker, J. K. (2017). Regional cerebral cortical thickness correlates with autonomic outflow. *Auton Neurosci.* 207, 28-36. doi: 10.1016/j.autneu.2017.05.012.

Xie, C., Bai, F., Yu, H., Shi, Y., Yuan, Y., Chen, G., et al. (2012). Abnormal insula functional network is associated with episodic memory decline in amnestic mild cognitive impairment. *Neuroimage*. 63, 320-327. doi: 10.1016/j.neuroimage.2012.06.062.

Yuan, P., and Raz, N. (2014). Prefrontal cortex and executive functions in healthy adults: a meta-analysis of structural neuroimaging studies. *Neurosci Biobehav Rev*. 42, 180-192. doi: 10.1016/j.neubiorev.2014.02.005.
